# Exosomal Thomsen–Friedenreich Glycoantigen as a Sensitive and Specific Biomarker for Colon, Ovarian and Prostate Cancer Diagnosis

**DOI:** 10.3390/cancers17233729

**Published:** 2025-11-21

**Authors:** Yafei Su, Man Qi, Shoaib Vasini, Mary E. Reid, Kate Rittenhouse-Olson, Grace K. Dy, Yun Wu

**Affiliations:** 1 Department of Biomedical Engineering, University at Buffalo, The State University of New York, Buffalo, NY 14260, USA; yafeisu@buffalo.edu (Y.S.); manqi@buffalo.edu (M.Q.); 2 Department of Electrical Engineering, University at Buffalo, The State University of New York, Buffalo, NY 14260, USA; 3 Department of Medicine, Roswell Park Comprehensive Cancer Center, Buffalo, NY 14203, USA; mary.reid@roswellpark.org; 4 For-Robin, Inc., Buffalo, NY 14221, USA; krolson@buffalo.edu

**Keywords:** thomsen–friedenreich glycoantigen, exosomes, extracellular vesicles, surface plasmon resonance, cancer liquid biopsy, colon cancer, ovarian cancer, prostate cancer, multi-cancer detection

## Abstract

**Background/Objectives:** The screening and diagnosis of colon, ovarian, and prostate cancers are challenged by invasive procedures and lack of effective screening tests. To address these challenges, we developed an exosome-based liquid biopsy assay that utilized a new exosomal biomarker, α-linked Thomsen–Friedenreich glycoantigen (TF-Ag-α, Galβ1-3GalNAc-α). This assay provides a minimally invasive method with high sensitivity and specificity for the screening and diagnosis of colon, ovarian and prostate cancers. **Methods**: Exosomal TF-Ag-α levels in 10 μL serum samples were measured using a compact, surface plasmon resonance-based biosensor developed to facilitate clinical translation. The diagnostic performance of exosomal TF-Ag-α was initially evaluated in a training set consisting of normal controls (*n* = 80) and cancer patients (*n* = 120, 40 for prostate, 40 for colon, and 40 for ovarian cancer), and then validated in an independent test set including normal controls (*n* = 29) and cancer patients (*n* = 60, 20 for each cancer type). Receiver operating characteristic (ROC) analysis was performed to assess diagnostic sensitivity, specificity and area under the curve (AUC) of exosomal TF-Ag-α. **Results**: Serum samples from cancer patients showed significantly elevated levels of exosomal TF-Ag-α compared with normal controls. Exosomal TF-Ag-α distinguished all cancer patients from normal controls with a sensitivity of 99.2%, specificity of 100% and an overall accuracy of 99.5% at a cutoff value of 0.406. **Conclusions**: These findings demonstrated the clinical translational promise of exosomal TF-Ag-α as a liquid biopsy biomarker for the detection of colon, ovarian and prostate cancers.

## Simple Summary

Current screening and diagnostic methods, such as colonoscopy for colon cancer, transvaginal ultrasound and the CA125 blood test followed by ovarian biopsy for ovarian cancer, and the prostate-specific antigen blood test and the digital rectal exam followed by prostate biopsy for prostate cancer, are often limited by invasiveness, suboptimal sensitivity and specificity, high costs, and poor patient compliance. There is an urgent need for accurate, non- or minimally invasive, and patient-friendly alternatives. In this study, we developed an exosome-based liquid biopsy test for colon, ovarian, and prostate cancers. We identified a novel exosomal biomarker, the alpha-linked Thomsen–Friedenreich glycoantigen (TF-Ag-α; Galβ1-3GalNAc-α), and demonstrated that exosomal TF-Ag-α is a highly sensitive and specific marker, capable of detecting colon, ovarian, and prostate cancers with 99.5% accuracy. This exosomal TF-Ag-α–based assay holds strong potential to complement existing tests, facilitate cancer screening and early detection, and ultimately improve patient outcomes.

## 1. Introduction

Cancer remains the leading cause of death worldwide, posing a significant public health challenge. Many malignancies, such as lung and ovarian cancers, are often diagnosed at advanced stages, where treatment options are limited and five-year survival rates are poor [1,2]. Early detection of cancer can dramatically improve outcomes, with survival rates increasing to 90% for some cancers [2,3,4]. Therefore, early cancer detection is critical for improving patient prognosis and the effectiveness of treatment. However, current screening and early detection methods for major cancers have notable limitations. For colon cancer, diagnostic methods include colonoscopy, fecal occult blood tests (FOBT), fecal immunochemical tests (FIT), and stool DNA tests, with CT colonography used in some cases [5]. Colonoscopy remains the gold standard, as it allows for direct visualization and biopsy of suspicious lesions. Nonetheless, it is invasive, requires bowel preparation and sedation, and carries a small risk of complications [6]. Stool-based tests, while less invasive, may miss early or precancerous lesions and often require follow-up colonoscopy if positive [7]. In the case of ovarian cancer, diagnosis typically relies on pelvic exams, transvaginal ultrasound, CA125 blood tests, and imaging such as CT scans, with definitive diagnosis often requiring surgical exploration [8]. However, symptoms of ovarian cancer are often nonspecific and usually present at a late stage; CA125 can be elevated in benign conditions; imaging is limited in detecting early-stage disease; and no effective screening test exists for the general population [8,9]. For prostate cancer, diagnostic approaches include prostate-specific antigen (PSA) blood tests, digital rectal exams (DRE), imaging (such as MRI), and confirmatory prostate biopsy [10]. PSA testing lacks specificity, leading to overdiagnosis and unnecessary biopsies; DRE is operator-dependent and may miss early tumors; and imaging and biopsy can be invasive [11,12]. New diagnostic approaches are urgently needed to overcome these limitations and enable earlier, more accurate detection across these cancer types.

One promising new approach to address these limitations is liquid biopsy, a minimally or non-invasive technique that analyzes tumor-derived materials—such as circulating tumor DNA (ctDNA), RNA, proteins, or exosomes—in bodily fluids like blood, urine, or saliva [13,14]. Liquid biopsy offers several advantages over traditional methods: it is less invasive, can be performed repeatedly to monitor disease progression or treatment response, and has the potential to detect cancers at earlier stages. Among the various components analyzed in liquid biopsies, tumor-derived exosomes have emerged as potent biomarkers for cancer liquid biopsy. Exosomes are small extracellular vesicles (sEVs) secreted by cells which carry molecular cargos, such as DNA, RNA, and proteins that reflect the physiological or pathological state of their cells of origin [15]. Because they are abundant in biofluids and contain rich molecular information, exosomes hold great promise for improving the sensitivity and specificity of cancer liquid biopsy across a wide range of cancer types, including colon, prostate, and ovarian cancers. For example, in colon cancer, elevated levels of exosomal miR-125a-3p in plasma were shown to distinguish early-stage patients from healthy individuals, with diagnostic accuracy improving when combined with carcinoembryonic antigen (CEA) [16,17]. For prostate cancer, tumor-derived exosomal αvβ3 integrin has been linked to aggressive disease and may serve as a non-invasive marker for monitoring progression [18,19]. In ovarian cancer, plasma-derived exosomal miR-4732-5p was significantly upregulated in patients, suggesting its utility as a promising biomarker for early detection [20]. These studies highlight exosome-based liquid biopsy as a powerful analytical assay capable of enabling minimally invasive and accurate cancer diagnosis.

Among various exosomal biomarkers, the Thomsen–Friedenreich glycoantigen (TF-Ag) has emerged as a promising candidate for cancer liquid biopsy. TF-Ag is the disaccharide D-galactose-β-(1–3)-N-acetylgalactosamine (Gal-β-(1–3)-GalNAc), which is typically α-linked to serine or threonine residues in normal tissues, but present in normal tissue as a more complex structure, either mono- or di-sialylated or otherwise further glycosylated by an N-acetylglucosamine addition to the galactose or by fucosylation. However, in tumor tissues, due to alterations in the glycosylation machinery, this disaccharide is present without further glycosylation [21]. This tumor-associated form, TF-Ag-α, is therefore a promising cancer-specific biomarker. In our previous study, we developed a TF-Ag-α–specific monoclonal antibody, JAA-F11, and demonstrated TF-Ag-α expression across a range of lethal human carcinomas, including breast, lung, colon, bladder, ovarian, prostate, and stomach cancers [22]. We further confirmed that TF-Ag-α is carried by exosomes and that serum exosomal TF-Ag-α could discriminate lung and breast cancers from normal controls with diagnostic accuracy of ≥95% and ≥97%, respectively [23]. Building on these findings, we aimed to evaluate the diagnostic potential of exosomal TF-Ag-α in colon, ovarian, and prostate cancers. Exosomal TF-Ag-α levels in serum samples were measured using a compact surface plasmon resonance (SPR) biosensor, designed to provide highly sensitive and rapid (~1 h) detection of exosomal biomarkers with minimal sample volume (as little as 10 μL of serum) and simple operation requiring only a micropipette. Exosomal TF-Ag-α levels were significantly higher in serum samples from patients with all examined cancer types (*n* = 60 for prostate, *n* = 60 for colon, and *n* = 60 for ovarian cancer) compared to normal controls (*n* = 109), achieving an overall accuracy of 99.5% at the cutoff value of 0.406. When combined with data from lung and breast cancer patients from our previous study, exosomal TF-Ag-α maintained a high discriminative accuracy of 96.2% across all five cancer types (prostate, colon, ovarian, lung, and breast), effectively distinguishing cancer patients (*n* = 335) from non-cancer controls (*n* = 187). These findings highlight the broad clinical utility of exosomal TF-Ag-α as a liquid biopsy biomarker for multi-cancer detection.

## 2. Materials and Methods

### 2.1. Materials

Thiolated biotin polyethylene glycol 1000 (Biotin-PEG1000-SH, MW 1000 Da, PG2-BNTH-1k) was purchased from Nanocs (New York, NY, USA). Methyl-PEG4-thiol (PEG200-SH, MW 224.32 Da, 26132), NeutrAvidin protein (31000), EZ-Link Micro Sulfo-NHS-LC-Biotinylation kit (21935), and glass microscope slides (12-550-A3) were purchased from Thermo Fisher Scientific (Waltham, MA, USA). JAA-F11 mouse antibodies (anti–TF-Ag-α mAb) were supplied by For-Robin, Inc (Buffalo, NY, USA). Mouse IgG3 antibodies (0105-01, RRID: AB_2793898) were purchased from SouthernBiotech (Birmingham, AL, USA).

### 2.2. Human Serum Sample Collection and Processing

Serum samples and corresponding clinical data were provided by the Data Bank and BioRepository Shared Resource at Roswell Park Comprehensive Cancer Center (Buffalo, NY, USA). Serum samples were collected from normal controls and treatment-naïve patients with colon, ovarian, or prostate cancer. The clinical data of all participants were de-identified. All participants provided informed consent prior to sample collection. All groups were matched on age, gender and race/ethnicity distributions. Serum samples were subsequently transferred to the University at Buffalo and stored at −80 °C. Prior to testing, serum samples were centrifuged at 10,000× *g* for 30 min at 4 °C to remove debris. The use of human serum samples in this study was approved by the Institutional Review Boards of Roswell Park Comprehensive Cancer Center and the University at Buffalo.

### 2.3. Fabrication of SPR Biochip

The SPR biochip fabrication was performed similarly to our prior work [23]. Briefly, glass slides were cleaned by sequential 10 min sonication in acetone, 200 proof ethanol, and deionized water. After cleaning, the slides underwent Ti (2 nm, 0.8 Å/s) and Au (49 nm, 0.8 Å/s) deposition by using an electron-beam evaporator (Kurt J. Lesker Company, Jefferson Hills, PA, USA). The biochip was assembled by bonding an Au-coated glass slide to a PDMS sheet with a 6 mm-diameter sample well after oxygen plasma treatment (forward RF power was 15 W, reflected power was 0 W, for 75 s).

### 2.4. Surface Modification of SPR Biochip

A 10 mmol/L thiolated polyethylene glycol (PEG) mixture was prepared by mixing Biotin-PEG1000-SH with PEG200-SH at a molar ratio of 1:3. The biochip surface was then coated with PEG via thiol–gold interactions by incubating the PEG mixture (100 μL) on the surface for 1 h at room temperature. After excess PEG was washed off using PBS, 100 μL of 50 μg/mL NeutrAvidin was added and immobilized on PEGylated biochip following 1 h incubation at room temperature. Finally, unbound NeutrAvidin was washed off with PBS, and 100 μL of 50 μg/mL biotinylated mouse JAA-F11 or mouse IgG3 antibodies was added and conjugated on the surface of biochip through the biotin–avidin interaction. The biochip was incubated at 4 °C overnight for future use.

### 2.5. Detection of Exosomal TF-Ag-α by the SPR Biosensor

The modified biochip was immobilized on the prism of the SPR biosensor. A 647 nm laser with a power density of 30 mW was directed through the sample well at the SPR angle. To measure the expression of exosomal TF-Ag-α, deionized water and PBS were first applied to the biochip, and the intensity of the reflected laser beam (I) was measured using a photodetector as baseline measurement. After acquiring the SPR signal for 2 min with both water (I_water_) and PBS (I_PBS_), 50 μL of diluted human serum sample (10 μL serum + 40 μL PBS) was added on the biochip and incubated for 1 h at room temperature. The TF-Ag-α-positive exosomes were captured by JAA-F11 antibodies, and the change in SPR signal was recorded. After washing off unbound exosomes, the SPR signal of TF-Ag-α-positive exosomes (I_exosomal TF-Ag-α_) was recorded for 2 min. The same sample was also applied on an IgG3 antibody modified biochip, which was used as a control for the measurement of expression of exosomal TF-Ag-α to remove background noise caused by nonspecific binding. The expression level of exosomal TF-Ag-α was calculated using the following equation:

$$\begin{array}{ll} Expression\;of & exosomal\;TF-Ag-\alpha \\ & ={\left(\frac{I_{exosomal\;TF-Ag-\alpha }-I_{PBS}}{I_{PBS}-I_{water}}\right)}_{JAA-F11\;antibody\;modified\;biochip}\\ & -{\left(\frac{I_{exosomal\;TF-Ag-\alpha }-I_{PBS}}{I_{PBS}-I_{water}}\right)}_{IgG3\;control\;antibody\;modified\;biochip} \end{array}$$

The difference between I_PBS_ and I_water_ was used as the normalization factor to address the chip-to-chip variation. Although each biochip was prepared using the same procedure, factors such as the thickness of gold film can vary slightly, which may affect the measured exosomal TF-Ag-α signals. While individual biochips may differ and the absolute values of I_PBS_ and I_water_ may vary, their difference remains consistent, reflecting the refractive index difference between water and PBS, and thus serves as the normalization factor to correct for chip-to-chip variation.

### 2.6. Characterization of Exosomes by Nanoparticle Tracking Analysis

The size, size distribution, and number concentration of exosomes isolated from patient serum samples were characterized using a nanoparticle tracking analysis (NTA) system (NanoSight, LM10; Malvern Panalytical, Westborough, MA, USA). Exosomes were first isolated from 100 μL of patient serum samples using a total exosome isolation kit (from serum; 4478360; Thermo Fisher Scientific), following the manufacturer’s instructions. The isolated exosomes were then diluted in PBS to achieve 50–100 nanoparticles within the field of view. All measurements were performed under identical instrument settings. Camera level, detection threshold, and screen gain were set at 14, 6, and 8, respectively, for view-capturing and particle tracking process.

### 2.7. Characterization of TF-Ag-α-Positive Tumor-Derived Exosomes

We employed scanning electron microscopy (SEM) to observe TF-Ag-α-positive, tumor-derived exosomes captured on the biochips. Briefly, after TF-Ag-α-positive exosomes were captured by JAA-F11 antibodies, unbounded exosomes were washed off using PBS. Then, the biochips were cleaned with deionized water and dried at 4 °C. Prior to SEM imaging, a layer of carbon was applied on the top of biochip, and TF-Ag-α-positive exosomes were characterized using a field-emission scanning electron microscope (SU-70; Hitachi High Technologies, Tokyo, Japan) with an energy-dispersive X-ray spectrometer.

### 2.8. Statistical Data Analysis

Receiver operating characteristic (ROC) analysis was performed using the SPSS software (Version 30; IBM, Armonk, NY, USA) to evaluate the discriminatory ability of exosomal TF-Ag-α between normal controls and cancer patients. In the training cohort, a logistic regression model was applied to generate the ROC curve, from which the area under the curve (AUC) was calculated and the optimal cutoff value was determined by the maximum Youden index (Youden Index = Sensitivity + Specificity − 1).

To assess diagnostic performance at the identified cutoff, a binary classification variable was created, and diagnostic metrics including sensitivity, specificity, positive predictive value (PPV), negative predictive value (NPV), and overall accuracy (ACC) were computed using the Crosstabs function in SPSS, based on the confusion matrix. The sensitivity was defined as the proportion of true positive cases among all actual positives, and specificity as the proportion of true negative cases among all actual negatives. PPV was calculated as the proportion of true positive cases among all test-positive samples, while NPV represented the proportion of true negative cases among all test-negative samples. ACC was calculated as the proportion of correctly classified cases (true positives and true negatives) among all samples.

The cutoff value identified from the training cohort was then applied to an independent test cohort. ROC analysis of the test cohort was performed using the observed exosomal TF-Ag-α values to generate the cross-validated AUC, and diagnostic metrics were calculated to evaluate classification performance.

## 3. Results

### 3.1. SPR-Based Detection and Characterization of Exosomal TF-Ag-α in Cancer Patient Serum

**Figure 1:**
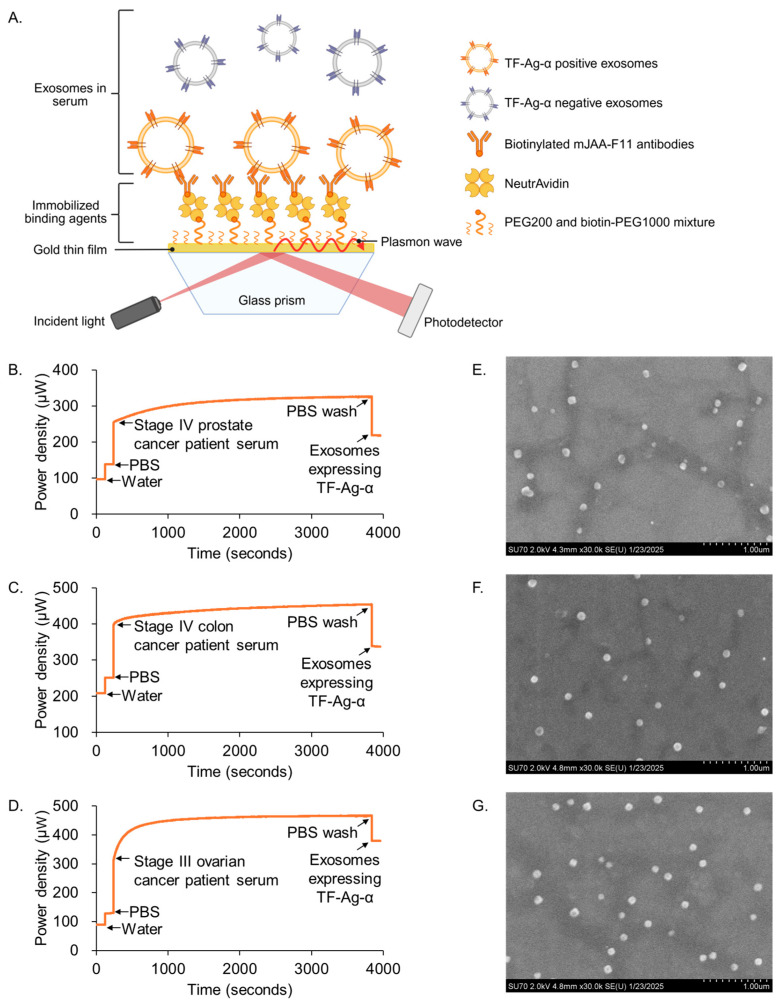
SPR assay detects exosomal TF-Ag-α for prostate, colon and ovarian cancer diagnosis. Schematic illustration of SPR-based detection of exosomal TF-Ag-α (**A**). Representative SPR curves for the detection of exosomal TF-Ag-α in serum samples from patients with prostate cancer (**B**), colon cancer (**C**), and ovarian cancer (**D**). SEM images of TF-Ag-α–positive exosomes captured on the biochips from serum samples of patients with prostate cancer (**E**), colon cancer (**F**), and ovarian cancer (**G**).

We employed a SPR-based liquid biopsy assay to assess the expression levels of exosomal TF-Ag-α in clinical serum samples from patients with prostate, colon, and ovarian cancers. The assay employed a compact SPR biosensor engineered for highly sensitive and fast (~1 h) detection of exosomal biomarkers. It requires only a small sample volume (as low as 10 μL of serum) and features a user-friendly design that can be operated with just a micropipette. In brief, the SPR assay detects changes in the local refractive index. As shown in Figure 1A, a laser beam is directed through a prism onto a gold-coated biochip, where JAA-F11 antibodies, specific for TF-Ag-α, are immobilized on the gold surface. Upon the addition of serum (10 µL serum diluted in 40 µL PBS) on the biochip, only exosomes expressing TF-Ag-α bind to the antibody-coated surface, inducing a shift in the refractive index. This change is then detected by a photodetector as a measurable SPR signal. Representative SPR sensing curves for detecting exosomal TF-Ag-α in serum samples from prostate, colon and ovarian cancer patients are shown in Figure 1B–D. To verify exosome capture on the biochips, SEM was performed. Representative SEM images (Figure 1E–G) confirmed the presence of exosomes from serum samples of prostate, colon, and ovarian cancer patients. The average size of exosomes on the biochips was 82.41 nm, which agreed with results from nanoparticle tracking analysis (Supporting Figure S1).

### 3.2. Evaluation of Exosomal TF-Ag-α in Colon Cancer Diagnosis

**Figure 2:**
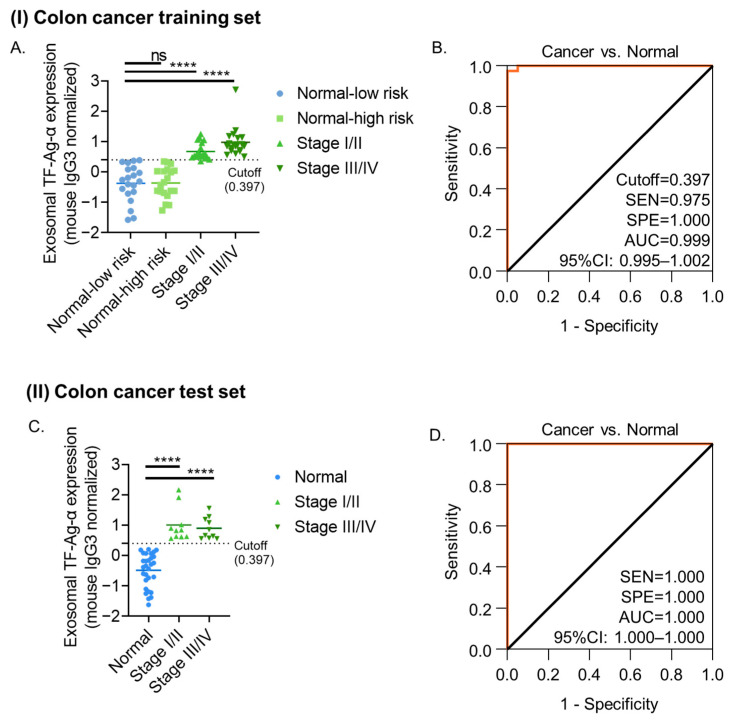
Evaluation of exosomal TF-Ag-α for colon cancer diagnosis using a training set and an independent test set. (**A**) Scatterplot showing serum exosomal TF-Ag-α levels in colon cancer patients (*n* = 40; stage I/II: *n* = 20; stage III/IV: *n* = 20) and normal controls (*n* = 40; low risk: *n* = 20; benign conditions at high risk of cancer: *n* = 20) in the training set. (**B**) ROC curve analysis comparing colon cancer patients vs. normal controls in the training set. (**C**) Scatterplot showing serum exosomal TF-Ag-α levels in colon cancer patients (*n* = 20; stage I/II: *n* = 10; stage III/IV: *n* = 10) and normal controls (*n* = 29) in the test set. (**D**) ROC curve analysis comparing colon cancer patients vs. normal controls in the test set. (SEN: sensitivity, SPE: specificity, AUC: area under the curve. ns: not significant; ****: *p* < 0.0001).

To evaluate the diagnostic performance of exosomal TF-Ag-α in colon cancer, a training set comprising patients with colon cancer (*n* = 40) and normal controls (*n* = 40) was established. The colon cancer group had all four stages of colon cancer (*n* = 10 at each stage). The normal control group included 20 low-risk individuals and 20 patients with benign colon conditions at high risk of colon cancer. The benign conditions included tubular adenoma, tubulovillous adenoma, villous adenoma, mucinous cystadenoma, diverticulosis, familial adenomatous polyposis, Gardner syndrome, abdominal desmoid tumors, and hereditary nonpolyposis colorectal cancer (HNPCC). Patient characteristics are summarized in Table 1 and Supplementary Table S1. The exosomal TF-Ag-α levels were significantly higher (*p* < 0.0001) in all colon cancer patients compared to both healthy individuals and those with benign colon conditions (Figure 2A). No significant difference was observed between healthy and benign control groups, nor among the different cancer stages, indicating that exosomal TF-Ag-α expression is not correlated with tumor burden or disease stage.

The diagnostic performance of exosomal TF-Ag-α in colon cancer was first evaluated by ROC analysis. Exosomal TF-Ag-α effectively distinguished colon cancer patients (*n* = 40) from normal controls (*n* = 40) with 97.5% sensitivity, 100% specificity, and an AUC of 0.999 at a cutoff value of 0.397 (Figure 2B). Additionally, confusion matrix analysis showed 97.5% sensitivity, 100% specificity, 100% PPV, 97.6% NPV, and 98.7% ACC at the cutoff value of 0.397, confirming the diagnostic performance (Supplementary Figure S2A). When stratified by stage, as shown in Supplementary Figure S2B,C, exosomal TF-Ag-α distinguished early-stage (I/II) colon cancer patients (*n* = 20) from controls with 95% sensitivity, 100% specificity, and an AUC of 0.998 at a cutoff value of 0.397. For late-stage (III/IV) patients (*n* = 20), both sensitivity and specificity reached 100%, with an AUC of 1.00 at a cutoff value of 0.442.

The diagnostic performance of exosomal TF-Ag-α in colon cancer was further validated in a blinded, independent test set analyzed by a different operator than the one who handled the training cohort. The test set included 20 colon cancer patients and 29 normal controls. Patient characteristics are provided in Table 1 and Supplementary Table S1. As shown in Figure 2C, exosomal TF-Ag-α levels were significantly higher (*p* < 0.0001) in serum samples from colon cancer patients compared to normal controls. Based on the observed exosomal TF-Ag-α levels, ROC analysis yielded a sensitivity of 100%, specificity of 100% and AUC of 1.00 (Figure 2D). Using the cutoff value of 0.397 derived from the training set, exosomal TF-Ag-α achieved 100% sensitivity, specificity, AUC, PPV, NPV, and ACC in distinguishing colon cancer patients (stages I–IV) from controls, surpassing the performance metrics observed in the training cohort (Supplementary Figure S2D–F). These results not only validated exosomal TF-Ag-α as a highly sensitive and specific biomarker for colon cancer diagnosis but also demonstrated the ease of use and operator-friendliness of the SPR assay, as highlighted in our previous study [23], supporting its strong potential for clinical application.

### 3.3. Diagnostic Evaluation of Exosomal TF-Ag-α in Ovarian Cancer

**Figure 3:**
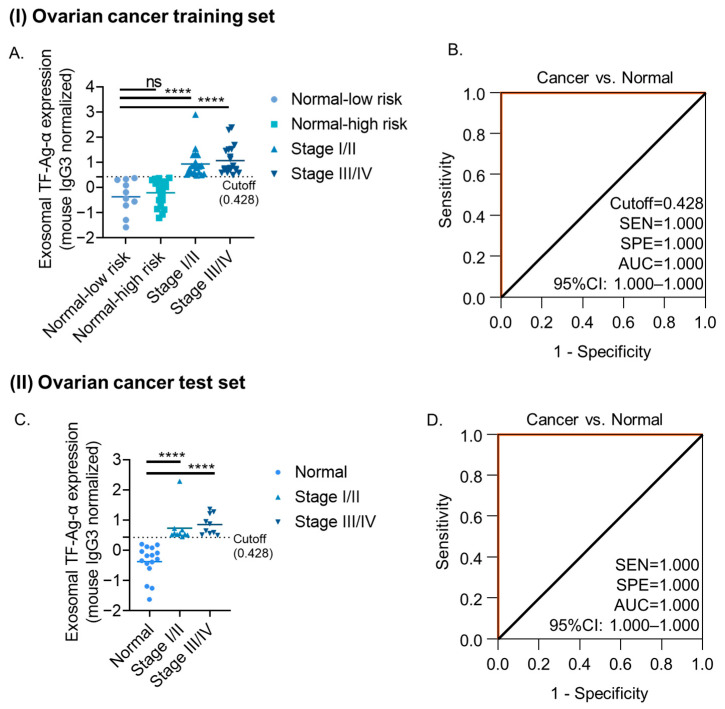
Evaluation of exosomal TF-Ag-α in ovarian cancer diagnosis using a training set and an independent test set. (**A**) Scatterplot of serum exosomal TF-Ag-α levels in ovarian cancer patients (*n* = 40; *n* = 10 for each stage) and normal controls (*n* = 30; low risk: *n* = 10; high risk: *n* = 20) in the training set. (**B**) ROC curve analyses comparing ovarian cancer patients vs. normal controls in the training set. (**C**) Scatterplot of exosomal TF-Ag-α levels in serum samples from ovarian cancer patients (*n* = 20; stage I/II: *n* = 10; stage III/IV: *n* = 10) and normal controls (*n* = 16) in the test set. (**D**) ROC curves comparing ovarian cancer patients and normal controls in the test set. (SEN: sensitivity, SPE: specificity, AUC: area under the curve. ns: not significant; ****: *p* < 0.0001.).

The diagnostic value of exosomal TF-Ag-α in ovarian cancer was first assessed using a training set consisting of ovarian cancer patients (*n* = 40) and normal controls (*n* = 30). The ovarian cancer group included 10 patients at each stage (stage I–IV), and the normal control group included 10 patients at low risk and 20 patients with benign ovarian conditions at high risk of ovarian cancer. The benign conditions included mucinous cystadenoma, serous cystadenoma, endometrioma, corpus luteum cyst, serous cyst, dermoid cyst, and follicular cyst. The patient characteristics are provided in Table 2 and Supplementary Table S2. Significantly higher levels of exosomal TF-Ag-α were detected (*p* < 0.0001) in ovarian cancer patients compared with low-risk controls and patients with benign ovarian conditions (Figure 3A). No significant difference in exosomal TF-Ag-α levels was observed between low-risk individuals and patients with benign ovarian conditions, and between early and late stages.

ROC analysis was conducted to evaluate the sensitivity, specificity, and AUC of exosomal TF-Ag-α in diagnosing ovarian cancer (Figure 3B). Exosomal TF-Ag-α successfully distinguished ovarian cancer patients (*n* = 40) from normal controls (*n* = 30) with 100% sensitivity, 100% specificity and AUC of 1.00 at cutoff value of 0.428. Confusion matrix analysis showed that the sensitivity, specificity, PPV, NPV, and ACC were 100% at the cutoff value of 0.428 (Supplementary Figure S3A). Exosomal TF-Ag-α also accurately differentiated both early-stage (I/II, *n* = 20) and late-stage (III/IV, *n* = 20) ovarian cancer patients from controls, with 100% sensitivity, 100% specificity and AUC of 1.00 at cutoff values of 0.428 and 0.437, respectively (Supplementary Figure S3B,C**)**.

The diagnostic performance of exosomal TF-Ag-α in ovarian cancer was validated using a blinded, independent test cohort consisting of 20 ovarian cancer patients and 16 normal controls. Patient characteristics are detailed in Table 2 and Supplementary Table S2. As shown in Figure 3C, exosomal TF-Ag-α levels were significantly elevated in serum samples from ovarian cancer patients compared to normal controls (*p* = 0.0002). Based on the observed exosomal TF-Ag-α levels, ROC analysis yielded a sensitivity, specificity, and AUC of 1.00 (Figure 3D). When applying the cutoff value of 0.428 established from the training set, exosomal TF-Ag-α achieved 100% sensitivity, specificity, AUC, PPV, NPV, and ACC in distinguishing ovarian cancer patients (stages I–IV) from normal controls (Supplementary Figure S3D–F). Results from both the training and test cohorts demonstrated that exosomal TF-Ag-α serves as a highly sensitive and specific biomarker for ovarian cancer diagnosis.

To evaluate whether exosomal TF-Ag-α outperforms the established biomarker CA125 in ovarian cancer diagnosis, we compared their diagnostic performance. Pre-treatment CA125 data were available for a subset of ovarian cancer patients (*n* = 27) and patients with benign conditions (*n* = 3), with blood samples collected within 100 days of those used for exosomal TF-Ag-α analysis to maintain temporal and analytical consistency. As shown in Supplementary Figure S4, at a cutoff value of 30.2 U/mL, CA125 demonstrated limited diagnostic accuracy, with a sensitivity of 74.1%, specificity of 33.3%, AUC of 0.728, NPV of 12.5%, PPV of 90.9%, and ACC of 70%. Among the 27 ovarian cancer patients, 18 were diagnosed at an early stage. The diagnostic performance of CA125 further declined when distinguishing early-stage ovarian cancer from benign conditions, yielding a sensitivity of 61.1%, specificity of 33.3%, AUC of 0.630, NPV of 12.5%, PPV of 84.6%, and ACC of 57.1%. In contrast, exosomal TF-Ag-α achieved perfect diagnostic performance in detecting ovarian cancer, including both early- and late-stage cases, with a sensitivity, specificity, AUC, NPV, PPV, and ACC all equal to 100%.

### 3.4. Evaluation of Exosomal TF-Ag-α in Prostate Cancer Diagnosis

**Figure 4:**
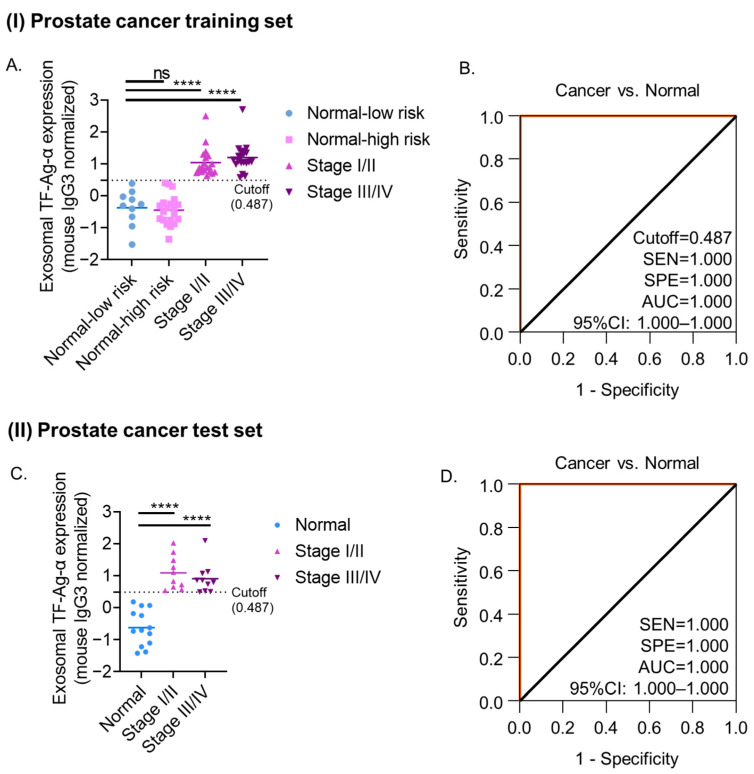
Evaluation of exosomal TF-Ag-α for prostate cancer diagnosis using a training set and an independent test set. (**A**) Scatterplot showing serum exosomal TF-Ag-α levels in prostate cancer patients (*n* = 40; stage I/II: *n* = 20; stage III/IV: *n* = 20) and normal controls (*n* = 30; low risk: *n* = 10; high risk: *n* = 20) in the training set. (**B**) ROC curve analysis comparing prostate cancer patients vs. normal controls in the training set. (**C**) Scatterplot showing serum exosomal TF-Ag-α levels in prostate cancer patients (*n* = 20; stage I/II: *n* = 10; stage III/IV: *n* = 10) and normal controls (*n* = 13) in the test set. (**D**) ROC curve analysis comparing prostate cancer patients vs. normal controls in the test set. (SEN: sensitivity, SPE: specificity, AUC: area under the curve. ns: not significant; ****: *p* < 0.0001.).

To evaluate the diagnostic performance of exosomal TF-Ag-α in prostate cancer, we first established a training set consisting of 40 prostate cancer patients and 30 normal controls. The prostate cancer group included 20 early-stage (I/II) and 20 late-stage (III/IV) patients. The normal control group included 10 patients at low risk and 20 patients with benign prostate conditions at high risk of prostate cancer. The benign conditions included benign prostatic hyperplasia (BPH), high-grade prostatic intraepithelial neoplasia (HGPIN, PIN III), focal glandular hyperplasia, benign phyllodes tumor, and prostatitis. The patient characteristics are provided in Table 3 and Supplementary Table S3. Exosomal TF-Ag-α levels were markedly elevated (*p* < 0.0001) in serum samples from prostate cancer patients (*n* = 40) compared to normal controls (*n* = 30) (Figure 4A). No significant difference was observed between low-risk controls (*n* = 10) and patients with benign prostate conditions (*n* = 20). Consistent with the findings in colon and ovarian cancers, exosomal TF-Ag-α levels did not differ significantly across prostate cancer stages, indicating that this biomarker is not associated with tumor progression.

ROC analysis revealed that exosomal TF-Ag-α could accurately discriminate prostate cancer patients (*n* = 40) from normal controls (*n* = 30), achieving 100% sensitivity, 100% specificity and an AUC of 1.00 at the cutoff value of 0.487 (Figure 4B). Confusion matrix analysis showed that the sensitivity, specificity, PPV, NPV, and ACC all reached 100% at the cutoff value of 0.487 (Supplementary Figure S5A). To further refine our assessment, prostate cancer patients were stratified into early-stage (stage I/II, *n* = 20) and late-stage (stage III/IV, *n* = 20) groups. Both groups were distinguished from normal controls with a sensitivity of 100%, specificity of 100% and AUC of 1.00 at cutoff values of 0.514 and 0.487, respectively (Supplementary Figure S5B,C). These findings demonstrated exosomal TF-Ag-α as a robust and potent biomarker for early-stage prostate cancer, capable of distinguishing prostate cancer patients from normal controls and those with benign prostate conditions.

The diagnostic performance of exosomal TF-Ag-α was validated in a blinded, independent test set of 20 prostate cancer patients (10 stage I/II and 10 state III/IV) and 13 normal controls (Table 3 and Supplementary Table S3). To assess reproducibility, measurements were independently performed by three operators. As shown in Figure 4C, serum exosomal TF-Ag-α levels were markedly higher in prostate cancer patients than in normal controls (*p* < 0.0001). ROC analysis of observed exosomal TF-Ag-α levels was conducted to compare overall prostate cancer patients versus normal controls, as well as early-stage (I/II) and late-stage (III/IV) subgroups versus normal controls (Figure 4D, Supplementary Figure S5E,F). In all three comparisons, the sensitivity and specificity were 100% and the AUC was 1.00, identical to results from the training cohort, demonstrating robust diagnostic accuracy. When the cutoff value of 0.487 identified from the training set was applied, exosomal TF-Ag-α achieved 100% sensitivity, specificity, AUC, PPV, NPV and ACC in distinguishing prostate cancer patients (stages I–IV) from normal controls (Supplementary Figure S5D). Notably, one sample from a stage I patient in test cohort, collected 75 days prior to clinical diagnosis, was correctly classified, further emphasizing the biomarker’s potential in early prostate cancer detection. These results demonstrate that exosomal TF-Ag-α is an ultra-sensitive and specific biomarker for prostate cancer detection, showing consistent performance across training and test cohorts.

To demonstrate that exosomal TF-Ag-α outperforms the conventional biomarker PSA in prostate cancer diagnosis, we compared their diagnostic performance. Pre-treatment PSA data were available for a subset of prostate cancer patients (*n* = 23) and patients with benign conditions (*n* = 5), with blood samples obtained within 100 days of those used for exosomal TF-Ag-α analysis to ensure comparability. As shown in Supplementary Figure S6, at a cutoff value of 4.0 ng/mL, PSA demonstrated poor diagnostic accuracy, with a sensitivity of 34.8%, specificity of 40.0%, AUC of 0.304, NPV of 11.8%, PPV of 72.7%, and ACC of 35.7%. When limited to early-stage prostate cancer cases (*n* = 8), PSA performance further deteriorated, with a sensitivity of 12.5%, specificity of 40.0%, AUC of 0.05, NPV of 22.2%, PPV of 25.0%, and ACC of 23.1%. In contrast, exosomal TF-Ag-α demonstrated outstanding diagnostic accuracy across both early- and late-stage prostate cancer, achieving 100% sensitivity, specificity, AUC, NPV, PPV, and ACC.

### 3.5. Evaluation of Exosomal TF-Ag-α Across Three Cancer Types (Colon, Ovarian, and Prostate)

**Figure 5:**
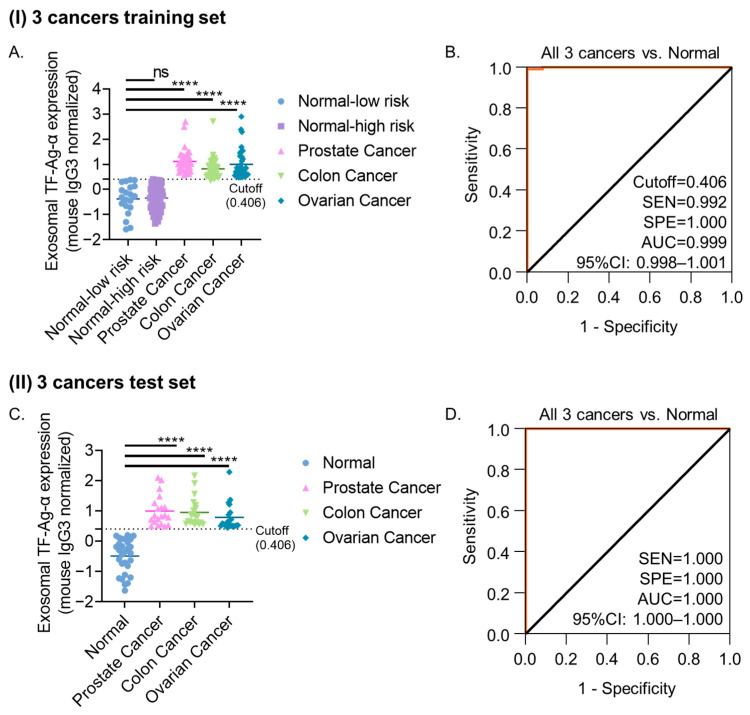
Evaluation of exosomal TF-Ag-α in three cancers’ diagnosis using the pooled training sets and independent test sets (colon, ovarian and prostate cancers). (**A**) Scatterplot showing serum exosomal TF-Ag-α levels in three cancer patients (*n* = 120; stage I/II: *n* = 60; stage III/IV: *n* = 60) and normal controls (*n* = 80; low risk: *n* = 20; high risk: *n* = 60) in the training sets. (**B**) ROC curve analysis comparing all 3 cancer patients vs. normal controls in the training set. (**C**) Scatterplot showing serum exosomal TF-Ag-α levels in three cancer patients (*n* = 60; stage I/II: *n* = 30; stage III/IV: *n* = 30) and normal controls (*n* = 29) in the test sets. (**D**) ROC curve analysis comparing all three cancer patients vs. normal controls in the test sets. (SEN: sensitivity, SPE: specificity, AUC: area under the curve. ns: not significant; ****: *p* < 0.0001).

After confirming the diagnostic performance of exosomal TF-Ag-α in detecting individual cancer types (prostate, colon, and ovarian cancer), we evaluated its diagnostic value across all three cancer types. We pooled the training sets for each cancer type, resulting in a combined cohort of 200 cancer patients and normal controls. As shown in Figure 5A, exosomal TF-Ag-α levels were significantly higher in cancer patients (*n* = 120) compared to non-cancer controls (*n* = 80; *n* = 20 at low risk, *n* = 60 at high risk) (*p* < 0.0001). Exosomal TF-Ag-α maintained high discriminative accuracy, yielding an AUC of 0.999 with 99.2% sensitivity and 100% specificity at a cutoff value of 0.406, as determined by ROC analysis (Figure 5B). When the cutoff value of 0.406 was applied for binary classification, confusion matrix analysis confirmed the diagnostic performance, yielding 99.2% sensitivity, 100% specificity, 100% PPV, 98.8% NPV, and 99.5% ACC (Supplementary Figure S7A). Exosomal TF-Ag-α distinguished normal controls from early-stage (I–II) cancer patients with 98.3% sensitivity, 100% specificity and an AUC of 0.999 at a cutoff value of 0.406, and from late-stage (III–IV) cancer patients with 100% sensitivity, 100% specificity and an AUC of 1.00 at a cutoff value of 0.448 (Supplementary Figure S7B–C).

Pooled analysis of all three cancer test cohorts (*n* = 89) confirmed significantly elevated exosomal TF-Ag-α levels in cancer patients (*n* = 60) compared with controls (*n* = 29) (*p* < 0.0001), with an AUC of 1.00, sensitivity of 100%, specificity of 100%, PPV of 100%, NPV of 100%, and ACC of 100% at a cutoff value of 0.406, outperforming the diagnostic performance observed in the training set (Figure 5C,D, Supplementary Figure S7D–F). Importantly, exosomal TF-Ag-α maintained high diagnostic accuracy in early-stage patients, underscoring its potential as a promising biomarker for early cancer detection.

### 3.6. Evaluation of Exosomal TF-Ag-α Across Five Cancer Types (Colon, Ovarian, Prostate, Lung and Breast)

**Figure 6:**
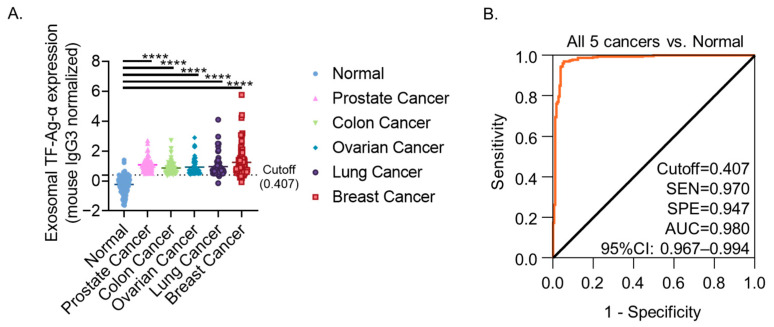
Evaluation of exosomal TF-Ag-α in 5 cancer diagnosis (colon, ovarian, prostate, lung and breast cancers). (**A**) Scatterplot showing serum exosomal TF-Ag-α levels in patients with five cancers (*n* = 335; stage 0/I/II: *n* = 174; stage III/IV: *n* = 161) and normal controls (*n* = 187; low risk: *n* = 89; high risk: *n* = 98). (**B**) ROC curve analysis comparing all cancer patients vs. normal controls. (SEN: sensitivity, SPE: specificity, AUC: area under the curve. ****: *p* < 0.0001).

Building on our previous findings in lung and breast cancers [23], pooled data from five cancer types (prostate, colon, ovarian, lung, and breast; total *n* = 522) were analyzed to assess the overall diagnostic performance of exosomal TF-Ag-α. Exosomal TF-Ag-α levels were significantly higher in cancer patients (*n* = 335; stage 0/I/II: *n* = 174; stage III/IV: *n* = 161) compared to non-cancer controls (*n* = 187; low risk: *n* = 89; benign condition at high risk: *n* = 98) (*p* < 0.0001; Figure 6A). Exosomal TF-Ag-α achieved high discriminative accuracy, with an AUC of 0.980, sensitivity of 97.0%, specificity of 94.7%, PPV of 97.0%, NPV of 94.7%, and ACC of 96.2% in distinguishing all cancer patients from normal controls at a cutoff value of 0.407 (Figure 6B, Supplementary Figure S8A). Exosomal TF-Ag-α analysis achieved high detection accuracy, with an AUC of 0.981, sensitivity of 97.6% and specificity of 94.7% in distinguishing early-stage cancer patients from normal controls at a cutoff value of 0.407, which underscores the potential of exosomal TF-Ag-α as an early cancer detection biomarker (Supplementary Figure S8B). Similarly, exosomal TF-Ag-α discriminated late-stage cancer patients from normal controls with an AUC of 0.979, sensitivity of 96.3% and specificity of 94.7% (Supplementary Figure S8C).

When analyzed by cancer type, a significant difference in exosomal TF-Ag-α levels was observed only between breast cancer and colon cancer (*p* = 0.0071), but not among other cancer types (Supplementary Figure S9A). No significant differences were detected across cancer stages (Supplementary Figure S9B,C). These findings indicate that exosomal TF-Ag-α levels remain largely consistent across cancer stages and exhibit only limited variation among tumor types. Unlike tissue-specific biomarkers that are limited to detecting individual cancer types, exosomal TF-Ag-α demonstrates consistent overexpression across diverse tumor origins. When combined with the high sensitivity, specificity, and label-free, real-time detection capabilities of SPR-based biosensors, this single-marker approach offers a promising platform for universal cancer screening independent of tissue origin.

## 4. Discussion

The United States Preventive Services Task Force (USPSTF) currently recommends screening tests for only four cancer types: low-dose CT scans for lung cancer, mammography for breast cancer, cytology/HPV testing for cervical cancer, and colonoscopy/stool-based testing for colorectal cancer [24,25,26,27]. While these screening modalities have demonstrated population-level benefits, they are constrained by significant limitations, including suboptimal sensitivity and specificity, invasive procedures, radiation exposure, and high cost. Moreover, many malignancies with lower prevalence rates, such as pancreatic and ovarian cancers, currently lack effective screening options [8,28]. This underscores an urgent need to develop highly sensitive, specific, and cost-effective screening methodologies for early cancer detection. Liquid biopsy platforms have emerged as particularly promising solutions, offering minimally invasive alternatives through the analysis of tumor-derived biomarkers in blood, urine, or other biofluids. Such approaches could potentially overcome key limitations of current methods by eliminating invasive procedures, reducing preparation complexity, and improving patient compliance.

Conventional blood-based biomarkers such as CA125 (ovarian cancer) and PSA (prostate cancer) remain in clinical use, yet their effectiveness for early detection is fundamentally constrained by insufficient sensitivity and specificity [8,29]. In contrast, exosomes represent a transformative biomarker class characterized by three biologically and clinically important properties: their active secretion from viable tumor cells, molecular cargos that recapitulate tumor biology, and abundant stable presence in circulation. These intrinsic features have established exosome-based diagnostics as a compelling platform across the cancer care continuum, from screening, early detection, treatment monitoring to prognosis.

We previously showed that TF-Ag-α is broadly expressed across multiple tumor types, including breast, lung, prostate, colon, bladder, and ovarian cancers but being absent in normal tissues, highlighting its diagnostic potential [22]. We further demonstrated that TF-Ag-α is present on exosomes, enabling accurate discrimination of lung and breast cancer patients from normal controls [23]. These findings inspired us to investigate the diagnostic utility of exosomal TF-Ag-α across additional cancer types. In the present study, we evaluated exosomal TF-Ag-α for the diagnosis of colon, ovarian, and prostate cancers using serum samples from 109 normal controls and 180 cancer patients. Employing our established SPR-based liquid biopsy platform with only 10 μL of serum per assay, we found that exosomal TF-Ag-α exhibited exceptional diagnostic performance, achieving 99.2% sensitivity, 100% specificity, and an AUC of 0.999 for distinguishing cancer patients from controls. Notably, exosomal TF-Ag-α maintained high detection capability for early-stage (I/II) cancers, with 98.3% sensitivity, 100% specificity, and an AUC of 0.999, demonstrating its strong potential for early cancer detection.

By integrating data from our previous and current studies (total *n* = 522; 335 patients with lung, breast, colon, ovarian, or prostate cancer and 187 normal controls), exosomal TF-Ag-α demonstrated strong diagnostic performance for multi-cancer detection. It distinguished all cancer patients from normal controls with a sensitivity of 97%, specificity of 94.7%, AUC of 0.980, NPV of 94.7%, PPV of 97% and ACC of 96.2%.

Our analysis revealed no significant differences in exosomal TF-Ag-α levels across cancer stages, suggesting this biomarker is not indicative of tumor progression stage. This observation may reflect inherent tumor heterogeneity, as we also observed that TF-Ag-α expression in tumor tissues was independent of disease stage. Our findings align with prior research by Takanami et al., who demonstrated that TF-Ag expression showed no correlation with gender, tumor size, lymph node involvement, distant metastasis, disease stage, or tumor differentiation grade in pulmonary adenocarcinoma. However, TF-Ag immunoreactivity did exhibit significant prognostic value for overall survival [30].

Although exosomal TF-Ag-α levels did not differ significantly among cancer stages, this limitation does not diminish its clinical utility for cancer screening and early detection. For a screening or diagnostic biomarker, the primary objective is to distinguish cancer from non-cancer individuals, particularly at early stages when intervention is most effective. In our subgroup analysis, exosomal TF-Ag-α demonstrated robust discrimination between stage 0/I/II cancers and normal controls, achieving an AUC of 0.981, sensitivity of 97.6%, and specificity of 94.7% across five cancer types. These findings indicate that exosomal TF-Ag-α holds great potential as a sensitive and reliable biomarker for cancer screening and early detection.

Among the five cancer types examined, only breast cancer exhibited significantly higher levels of exosomal TF-Ag-α compared to colon cancer, with no significant differences observed among the other cancers. These findings indicate that exosomal TF-Ag-α alone has limited ability to distinguish between cancer types. However, it can be incorporated into a multianalyte test alongside organ- or tumor type-specific biomarkers to enable simultaneous detection and localization of multiple cancer types. Moreover, its high sensitivity and specificity for early-stage disease make it an attractive candidate for inclusion in multimodal screening strategies. For example, exosomal TF-Ag-α may serve as a complementary blood test to low dose CT for lung cancer, as an adjunctive triage test for mammography in breast cancer, and as an alternative/triage modality alongside colonoscopy/stool-based testing for colorectal cancer.

Collectively, our results have demonstrated that exosomal TF-Ag-α could serve as a robust and broadly applicable biomarker for early multi-cancer detection. Moving forward, we will evaluate its diagnostic performance in additional cancer types, such as brain and bladder cancers. We plan to further validate the clinical utility of exosomal TF-Ag-α in large patient cohorts. For example, our comparison with PSA indicates that exosomal TF-Ag-α demonstrates substantially superior diagnostic performance for prostate cancer. However, this comparison was limited to a small subset of patients and lacked comprehensive clinical annotations, including Gleason scores, molecular data, and longitudinal outcomes. Thus, although exosomal TF-Ag-α appears more accurate than PSA for detecting prostate cancer, further studies are needed to determine whether it preferentially identifies higher-risk disease or reduces overtreatment of indolent tumors. Prospective studies incorporating detailed histopathologic and genomic information will be essential to evaluate whether exosomal TF-Ag-α correlates with tumor aggressiveness and clinical progression.

To assess its value for early cancer detection, we plan to collect blood samples from patients at various time points prior to diagnosis to determine how early exosomal TF-Ag-α can indicate the presence of cancer. We will also investigate whether exosomal TF-Ag-α can serve as a complementary assay to enhance the accuracy of existing screening and diagnostic methods. For example, in cases where low-dose CT detects an indeterminate nodule, measuring exosomal TF-Ag-α levels may help distinguish between benign and malignant findings. Additionally, combining exosomal TF-Ag-α with established markers such as CA125 for ovarian cancer and PSA for prostate cancer could improve diagnostic accuracy.

Given that elevated exosomal TF-Ag-α levels are observed in multiple cancer types, it may be used in conjunction with cancer-type- or organ-specific markers to develop a multi-cancer early detection (MCED) test. Furthermore, exosomal TF-Ag-α holds promise as a biomarker for monitoring treatment response, particularly for procedures involving organ removal. For instance, following mastectomy for breast cancer, few effective methods exist to monitor recurrence [31,32,33,34]. Tracking changes in exosomal TF-Ag-α expression may offer a valuable approach for early relapse detection.

## 5. Conclusions

In summary, we showed that exosomal TF-Ag-α was a highly sensitive and specific biomarker for colon, ovarian, and prostate cancers. The exosome-based liquid biopsy assay achieved 99.5% diagnostic accuracy across all three cancer types. This approach offers a simple, accurate, and minimally invasive alternative to current diagnostic procedures, with the potential to enhance current diagnostic practices, enable early cancer detection, and ultimately improve patient outcomes.

## Figures and Tables

**Table 1:** Characteristics of colon cancer patients and normal controls.

Characteristics *n* (%)				
	Training set		Test set	
Total patients	80		49	
Normal controls (low risk)	20 (25)		29 (59)	
Normal controls (high risk)	20 (25)		0 (0)	
Cancer	40 (50)		20 (41)	
	Normal controls (both low-risk and high-risk patients)	Patients with colon cancer	Normal controls (low-risk patients)	Patients with colon cancer
Gender				
Female	20 (25)	20 (25)	16 (33)	10 (20)
Male	20 (25)	20 (25)	13 (27)	10 (20)
Age (years)				
Mean	60	65	64	61
Median	60	66	64	58
Range	30–79	44–91	50–79	45–79
Stage				
I		10 (25)		5 (25)
II		10 (25)		5 (25)
III		10 (25)		5 (25)
IV		10 (25)		5 (25)

**Table 2:** Characteristics of ovarian cancer patients and normal controls.

Characteristics *n* (%)				
	Training set		Test set	
Total patients	70		36	
Normal controls (low risk)	10 (14)		16 (44)	
Normal controls (high risk)	20 (29)		0 (0)	
Cancer	40 (57)		20 (56)	
	Normal controls (both low-risk and high-risk patients)	Patients with ovarian cancer	Normal controls (low-risk patients)	Patients with ovarian cancer
Gender				
Female	30 (43)	40 (57)	16 (44)	20 (56)
Age (years)				
Mean	58	65	64	59
Median	57	65	66	60
Range	38–85	40–89	50–79	47–76
Stage				
I		10 (25)		5 (25)
II		10 (25)		5 (25)
III		10 (25)		5 (25)
IV		10 (25)		5 (25)

**Table 3:** Characteristics of prostate cancer patients and normal controls.

Characteristics *n* (%)				
	Training set		Test set	
Total patients	70		33	
Normal controls (low risk)	10 (14)		13 (39)	
Normal controls (high risk)	20 (29)		0 (0)	
Cancer	40 (57)		20 (61)	
	Normal controls (both low-risk and high-risk patients)	Patients with prostate cancer	Normal controls (low-risk patients)	Patients with prostate cancer
Gender				
Male	30 (43)	40 (57)	13 (39)	20 (61)
Age (years)				
Mean	64	60	63	62
Median	64	60	61	59
Range	51–78	45–81	51–77	53–74
Stage				
I		10 (25)		5 (25)
II		10 (25)		5 (25)
III		10 (25)		5 (25)
IV		10 (25)		5 (25)

## Data Availability

All key data are included in the Supporting Information. Additional data will be made available by the authors upon reasonable request.

## Supplementary Materials

The following supporting information can be downloaded at: https://www.mdpi.com/article/10.3390/cancers17233729/s1, Figure S1: Characterization of exosomes isolated from patient serum samples via nanoparticle tracking analysis (NTA); Figure S2: Evaluation of exosomal TF-Ag-α for colon cancer diagnosis using a training set and an independent test set; Figure S3: Evaluation of exosomal TF-Ag-α for ovarian cancer diagnosis using a training set and an independent test set; Figure S4: Diagnostic performance comparison between CA125 and exosomal TF-Ag-α in ovarian cancer; Figure S5: Evaluation of exosomal TF-Ag-α for prostate cancer diagnosis using a training set and an independent test set; Figure S6: Diagnostic performance comparison between PSA and exosomal TF-Ag-α in prostate cancer; Figure S7: Evaluation of exosomal TF-Ag-α in three cancer diagnosis using the pooled training sets and independent test sets (colon, ovarian and prostate cancers); Figure S8: Evaluation of exosomal TF-Ag-α in five cancer diagnosis (colon, ovarian, prostate, lung and breast cancers); Figure S9: Exosomal TF-Ag-α levels remain largely consistent across cancer types and stages; Table S1: Characteristics of Colon Cancer Patients and Controls; Table S2: Characteristics of Ovarian Cancer Patients and Controls; Table S3: Characteristics of Prostate Cancer Patients and Controls.

## Abbreviations

The following abbreviations are used in this manuscript:

ACC	Overall accuracy
AUC	Area under the curve
BPH	Benign prostatic hyperplasia
CEA	Carcinoembryonic antigen
ctDNA	Circulating tumor DNA
DRE	Digital rectal exam
FIT	Fecal immunochemical tests
FOBT	Fecal occult blood test
HGPIN	High-grade prostatic intraepithelial neoplasia
HNPCC	Hereditary nonpolyposis colorectal cancer
MCED	Multi-cancer early detection
MRI	Magnetic resonance imaging
NPV	Negative predictive value
NTA	Nanoparticle tracking analysis
PEG	Polyethylene glycol
PPV	Positive predictive value
PSA	Prostate-specific antigen
ROC	Receiver operating characteristic
SEM	Scanning electron microscopy
SEN	Sensitivity
sEV	Small extracellular vesicle
SPE	Specificity
SPR	Surface plasmon resonance
TF-Ag-α	Alpha-linked Thomsen–Friedenreich glycoantigen
USPSTF	United States Preventive Services Task Force
