# Supplementary material for: Exosomal Thomsen–Friedenreich Glycoantigen as a Sensitive and Specific Biomarker for Colon, Ovarian and Prostate Cancer Diagnosis
Source: Cancers (Basel). 2025 Nov 21;17(23):3729. doi: 10.3390/cancers17233729 (PMC12691054; doi:10.3390/cancers17233729)
Supplement: Supplementary file 1 [file cancers-17-03729-s001.zip › cancers-3874206-supplementary.pdf]

cancers

MDPI

Supporting Information

Exosomal Thomsen–Friedenreich Glycoantigen as a Sensitive and Specific Biomarker for Colon, Ovarian and Prostate Cancer Diagnosis

Yafei Su [1], Man Qi [1], Shoaib Vasini [2], Mary E. Reid [3], Kate Rittenhouse-Olson [4], Grace K. Dy [3,]*and Yun Wu [1,]*

[1] Department of Biomedical Engineering, University at Buffalo, The State University of New York, Buffalo, New York, 14260, USA; YS: yafeisu@buffalo.edu; MQ: manqi@buffalo.edu; YW: ywu32@buffalo.edu
[2] Department of Electrical Engineering, University at Buffalo, The State University of New York, Buffalo, New York, 14260, USA; shoaibva@buffalo.edu
[3] Department of Medicine, Roswell Park Comprehensive Cancer Center, Buffalo, New York, 14203, USA; MER: mary.reid@roswellpark.org; GKD: grace.dy@roswellpark.org
[4] For-Robin, Inc., Buffalo, New York, 14221, USA; krolson@buffalo.edu
* Correspondence: GKD: grace.dy@roswellpark.org; Tel.: (716) 845-3099; YW: ywu32@buffalo.edu; Tel.: (716)645-8498

 https://doi.org/10.3390/cancers17233729

Supplementary Table S1. Characteristics of Colon Cancer Patients and Controls

ID	Cancer Morphology	Race	Gender	Age	Stage	Exosomal TF-Ag-α level (A.U.)
**Training set (40 normal controls and 40 cancer cases)**						
1	Normal control	White	F	65		0.37
2	Normal control	White	M	59		-0.27
3	Normal control	White	F	70		0.33
4	Normal control	White	F	50		-0.19
5	Normal control	Black	F	70		-0.72
6	Normal control	Black	F	60		0.08
7	Normal control	White	M	76		0.13
8	Normal control	White	F	77		-0.56
9	Normal control	White	M	52		-0.41
10	Normal control	Black	M	60		-0.66
11	Normal control	White	F	79		-1.59
12	Normal control	White	F	53		0.30
13	Normal control	White	F	68		-0.45
14	Normal control	White	M	57		-0.03
15	Normal control	White	M	69		0.38
16	Normal control	White	F	65		-1.30
17	Normal control	Black	M	69		-0.96
18	Normal control	White	M	62		-0.31
19	Normal control	White	M	66		-0.12
20	Normal control	White	M	51		-1.53
21	Tubular Adenoma	White	F	52	Benign	0.08
22	Diverticular disease, diverticulosis	White	F	62	Benign	-0.62
23	Diverticular disease, diverticulosis	White	M	79	Benign	-0.69
24	Tubular Adenoma	White	F	63	Benign	-0.63
25	Familial Adenomatous polyposis	White	F	51	Benign	-0.43
26	Tubulovillous Adenoma	White	M	73	Benign	0.27
27	Diverticular disease, diverticulosis	White	M	60	Benign	0.04
28	Gardner Syndrome	White	M	57	Benign	-0.65
29	Mucinous Cystadenoma	White	F	47	Benign	0.02
30	Polyposis Coli	White	M	44	Benign	-0.01
31	Abdominal desmoid tumors	White	M	68	Benign	0.35
32	Familial Adenomatous	White	M	47	Benign	-1.09

	polyposis					
33	Tubulovillous Adenoma	White	F	70	Benign	-0.63
34	Adenomatous polyp	Black	M	53	Benign	0.32
35	Hereditary nonpolyposis colorectal cancer (HNPCC)	White	F	47	Benign	-1.10
36	Polyp of colon	White	M	58	Benign	-0.34
37	HNPCC	White	F	46	Benign	-1.27
38	Villous Adenoma	White	F	30	Benign	-0.21
39	Mucinous tumor of low malignant potential	White	M	63	Benign	-0.79
40	Adenomatous polyp	White	F	60	Benign	0.02
41	Adenocarcinoma, NOS	White	F	80	1	0.56
42	Adenocarcinoma, NOS	White	F	50	1	0.96
43	Adenocarcinoma, NOS	Black	M	64	1	1.12
44	Adenocarcinoma, NOS	White	M	51	1	0.50
45	Adenocarcinoma in adenomatous polyp	Unknown	F	51	1	0.36
46	Adenocarcinoma, NOS	White	F	47	1	0.79
47	Adenocarcinoma, NOS	White	M	74	1	0.56
48	Adenocarcinoma, NOS	White	M	56	1	1.06
49	Adenocarcinoma in tubular adenoma	White	M	53	1	0.56
50	Adenocarcinoma, NOS	White	F	77	1	0.52
51	Adenocarcinoma, NOS	White	M	68	2A	0.45
52	Adenocarcinoma, NOS	White	F	60	2A	0.41
53	Adenocarcinoma, NOS	White	M	91	2A	0.48
54	Adenocarcinoma, NOS	White	F	49	2A	0.53
55	Adenocarcinoma, NOS	White	F	80	2A	0.49
56	Adenocarcinoma, NOS	White	F	87	2A	1.05
57	Gastrointestinal stromal sarcoma	White	M	74	2B	0.52
58	Adenocarcinoma, NOS	White	M	67	2C	0.65
59	Mucinous adenocarcinoma	White	F	71	2C	0.61
60	Mucinous adenocarcinoma	White	F	82	2C	1.25
61	Adenocarcinoma, NOS	Unknown	M	59	3B	0.56
62	Adenocarcinoma, NOS	White	M	65	3B	0.85
63	Adenocarcinoma, NOS	White	F	84	3B	0.85
64	Adenocarcinoma, NOS	Native American	F	44	3B	0.78
65	Mucinous	White	M	68	3B	0.60

	adenocarcinoma					
66	Adenocarcinoma, NOS	White	M	65	3C	0.90
67	Adenocarcinoma in adenomatous polyp	White	F	59	3A	0.70
68	Adenocarcinoma in tubulovillous adenoma	White	M	53	3A	0.50
69	Adenocarcinoma, NOS	White	M	56	3B	1.14
70	Adenocarcinoma, NOS	White	F	67	3B	2.71
71	Adenocarcinoma in tubulovillous adenoma	White	M	85	4	0.93
72	Signet ring cell carcinoma	Unknown	F	50	4	1.24
73	Mucinous adenocarcinoma	White	M	66	4A	0.71
74	Adenocarcinoma, NOS	White	F	62	4A	0.91
75	Adenocarcinoma, NOS	White	F	69	4A	0.85
76	Adenocarcinoma, NOS	White	M	63	4B	1.18
77	Adenocarcinoma, NOS	White	M	69	4B	0.91
78	Small cell carcinoma, NOS	White	M	67	4B	1.37
79	Mucinous adenocarcinoma	White	F	67	4C	0.68
80	Adenocarcinoma, NOS	White	F	55	4C	1.11
**Test set (29 normal controls and 20 cancer cases)**						
81	Normal control (low risk)	White	F	50		-0.09
82	Normal control (low risk)	White	M	54		0.07
83	Normal control (low risk)	White	F	70		-0.04
84	Normal control (low risk)	Black	F	66		-0.18
85	Normal control (low risk)	Black	M	58		-1.11
86	Normal control (low risk)	Black	M	51		-0.81
87	Normal control (low risk)	Asian	M	54		-1.23
88	Normal control (low risk)	Black	F	56		-0.29
89	Normal control (low risk)	White	F	55		-1.26
90	Normal control (low	White	M	72		-0.63

	risk)					
91	Normal control (low risk)	White	M	67		-1.39
92	Normal control (low risk)	White	F	72		-0.35
93	Normal control (low risk)	White	M	77		-0.71
94	Normal control (low risk)	White	M	72		-1.43
95	Normal control (low risk)	White	F	57		0.12
96	Normal control (low risk)	White	F	77		-0.44
97	Normal control (low risk)	White	F	52		0.18
98	Normal control (low risk)	White	F	77		-1.63
99	Normal control (low risk)	White	M	55		-0.74
100	Normal control (low risk)	White	F	69		0.20
101	Normal control (low risk)	White	M	72		0.18
102	Normal control (low risk)	White	M	72		-0.25
103	Normal control (low risk)	White	F	58		-0.60
104	Normal control (low risk)	White	M	61		-0.19
105	Normal control (low risk)	White	M	55		0.07
106	Normal control (low risk)	White	F	60		-1.20
107	Normal control (low risk)	White	F	67		-0.39
108	Normal control (low risk)	White	F	79		0.08
109	Normal control (low risk)	Asian	F	69		-0.16
110	Adenocarcinoma in villous adenoma	White	F	45	1	1.01
111	Adenocarcinoma, NOS	White	F	74	1	0.62
112	Adenocarcinoma in	White	M	57	1	0.62

	adenomatous polyp					
113	Adenocarcinoma, NOS	Black	M	61	1	0.90
114	Adenocarcinoma in tubulovillous adenoma	White	F	50	1	0.84
115	Adenocarcinoma in polypoid adenoma	White	F	79	2A	1.91
116	Adenocarcinoma in tubulovillous adenoma	White	M	54	2A	0.56
117	Adenocarcinoma, NOS	White	M	74	2A	2.17
118	Adenocarcinoma, NOS	White	F	58	2A	0.82
119	Adenocarcinoma with mixed subtypes	White	F	68	2B	0.63
120	Adenocarcinoma in tubulovillous adenoma	White	F	56	3A	0.56
121	Adenocarcinoma, NOS	White	F	55	3B	0.56
122	Adenocarcinoma, NOS	White	M	69	3C	0.64
123	Adenocarcinoma, NOS	White	M	66	3C	1.56
124	Adenocarcinoma, NOS	White	M	56	3C	1.28
125	Adenocarcinoma, NOS	White	M	56	4	0.86
126	Adenocarcinoma in tubulovillous adenoma	White	F	52	4A	0.66
127	Adenocarcinoma in tubular adenoma	White	M	63	4A	0.58
128	Adenocarcinoma, NOS	White	M	65	4B	1.09
129	Adenocarcinoma, NOS	White	F	52	4C	1.19

Supplementary Table S2. Characteristics of Ovarian Cancer Patients and Controls

ID	Cancer Morphology	Race	Gender	Age	Stage	Exosomal TF-Ag-α level (A.U.)	Serum CA125 level (U/mL)
**Training set (30 normal controls and 40 cancer cases)**							
*1	Normal control	White	F	65		0.37	N/A
*2	Normal control	White	F	70		0.33	N/A
*3	Normal control	White	F	50		-0.19	N/A
*4	Normal control	Black	F	70		-0.72	N/A
*5	Normal control	Black	F	60		0.08	N/A
*6	Normal control	White	F	77		-0.56	N/A
*7	Normal control	White	F	79		-1.59	N/A
*8	Normal control	White	F	53		0.30	N/A
*9	Normal control	White	F	68		-0.45	N/A
*10	Normal control	White	F	65		-1.30	N/A
11	Dermoid Cyst	White	F	41	Benign	0.34	N/A
12	Mucinous Cystade-noma	White	F	56	Benign	0.20	N/A
13	Mucinous Cystade-noma	White	F	62	Benign	0.37	N/A
14	Corpus Luteum Cyst	White	F	47	Benign	-1.08	N/A
15	Serous Cystadenoma	White	F	60	Benign	-0.30	N/A
16	Serous Cystadenoma	White	F	53	Benign	-0.74	N/A
17	Serous Cyst	White	F	57	Benign	-0.25	N/A
18	Corpus Luteum Cyst	White	F	47	Benign	-0.86	N/A
19	Cystadenoma	White	F	54	Benign	0.02	N/A
20	Serous Cyst	White	F	65	Benign	0.38	N/A
21	Mucinous Cystade-noma	White	F	56	Benign	-0.51	N/A
22	Endometrioma	White	F	38	Benign	0.23	N/A
23	Serous Cystadenoma	White	F	54	Benign	-0.88	N/A
24	Serous Cyst	White	F	85	Benign	-0.51	N/A
25	Endometrioma	White	F	47	Benign	0.15	64.10
26	Dermoid Cyst	White	F	40	Benign	0.23	12.40
27	Follicular Cyst	Black	F	45	Benign	0.29	31.30
28	Cystadenoma	White	F	69	Benign	-0.01	N/A
29	Serous Cystadenoma	White	F	61	Benign	-1.21	N/A
30	Follicular Cyst	White	F	52	Benign	-0.08	N/A
31	Serous cystadenoma, borderline malig	White	F	67	1A	0.60	7.3

32	Endometrioid adenocarcinoma, NOS	White	F	54	1A	0.87	11.0
33	Carcinosarcoma, NOS	White	F	73	1A	0.83	9.0
34	Papillary transitional cell carcinoma	White	F	65	1A	0.51	4.8
35	Serous surface papillary carcinoma (C56.9)	White	F	63	1B	1.29	7.5
36	Clear cell carcinoma	White	F	52	1C	0.56	46.6
37	Mucinous cystadenocarcinoma, NOS	White	F	57	1C	0.54	211.3
38	Mucinous carcinoma	White	F	70	1C	0.61	30.1
39	Adult granulosa cell tumor of ovary	White	F	68	1C	0.48	N/A
40	Mucinous cystadenocarcinoma, NOS	White	F	54	1C	1.29	160.3
41	Adenocarcinoma, NOS	White	F	53	2A	0.85	99.8
42	Serous surface papillary carcinoma (C56.9)	White	F	84	2A	1.54	23.6
43	Serous adenocarcinoma, NOS (C56.9)	White	F	77	2A	0.86	333.1
44	Mixed cell adenocarcinoma	White	F	55	2B	1.33	211.8
45	Serous carcinoma, NOS	Black	F	59	2C	1.00	102.2
46	Serous carcinoma, NOS	White	F	60	2C	0.63	23.3
47	Papillary serous cystadenocarcinoma	White	F	40	2C	0.78	9.5
48	Clear cell adenocarcinoma, NOS	White	F	67	2B	2.90	N/A
49	Clear cell adenocarcinoma, NOS	White	F	55	2B	0.73	33.5
50	Serous surface papillary carcinoma (C56.9)	White	F	77	2B	0.49	54.0
51	Serous surface papillary carcinoma (C56.9)	White	F	60	3B	0.58	N/A
52	Serous surface papillary carcinoma (C56.9)	White	F	68	3C	1.53	N/A
53	Carcinosarcoma, NOS	White	F	67	3C	0.49	5.1
54	Serous cystadenocarcinoma, NOS (C56.9)	White	F	58	3C	1.69	107.0
55	Serous surface papillary carcinoma	White	F	82	3C	1.14	N/A

56	Serous surface papillary carcinoma (C56.9)	White	F	76	3C	2.39	167.6
57	Mixed cell adenocarcinoma	White	F	89	3C	0.70	N/A
58	Mixed cell adenocarcinoma	White	F	75	3C	0.59	74.7
59	Serous surface papillary carcinoma	White	F	59	3C	0.58	12.1
60	Serous surface papillary carcinoma (C56.9)	White	F	76	3C	0.73	131.1
61	High grade serous carcinoma	White	F	65	4	0.70	N/A
62	Mixed cell adenocarcinoma	White	F	61	4	1.45	30.4
63	Papillary serous cystadenocarcinoma	White	F	68	4	0.69	165.6
64	Mixed cell adenocarcinoma	White	F	54	4	1.22	104.4
65	Serous cystadenocarcinoma, NOS (C56.9)	White	F	62	4	0.77	12.3
66	Papillary serous cystadenocarcinoma	White	F	68	4	1.51	9.2
67	Mesodermal mixed tumor	White	F	78	4	0.86	2847.3
68	Serous surface papillary carcinoma (C56.9)	White	F	77	4	0.73	218.9
69	Serous surface papillary carcinoma (C56.9)	White	F	65	4	0.75	361.8
70	Serous surface papillary carcinoma	White	F	51	4B	2.29	8399.2
**Test set (16 normal controls and 20 cancer cases)**							
*71	Normal control (low risk)	White	F	50		-0.09	N/A
*72	Normal control (low risk)	White	F	70		-0.04	N/A
*73	Normal control (low risk)	Black	F	66		-0.18	N/A
*74	Normal control (low risk)	Black	F	56		-0.29	N/A
*75	Normal control (low risk)	White	F	55		-1.26	N/A
*76	Normal control (low risk)	White	F	72		-0.35	N/A

*77	Normal control (low risk)	White	F	57		0.12	N/A
*78	Normal control (low risk)	White	F	77		-0.44	N/A
*79	Normal control (low risk)	White	F	52		0.18	N/A
*80	Normal control (low risk)	White	F	77		-1.63	N/A
*81	Normal control (low risk)	White	F	69		0.20	N/A
*82	Normal control (low risk)	White	F	58		-0.60	N/A
*83	Normal control (low risk)	White	F	60		-1.20	N/A
*84	Normal control (low risk)	White	F	67		-0.39	N/A
*85	Normal control (low risk)	White	F	79		0.08	N/A
*86	Normal control (low risk)	Asian	F	69		-0.16	N/A
87	Mixed cell adenocarcinoma	White	F	57	1A	0.55	7.0
88	Endometrioid adenocarcinoma, NOS	White	F	60	1A	0.53	9.4
89	Mixed cell adenocarcinoma	White	F	48	1C	0.45	19.6
90	Serous tumor, NOS, low malignant potential	White	F	48	1C	0.51	45.6
91	Serous cystadenocarcinoma, NOS (C56.9)	White	F	67	1C	0.67	111.7
92	Endometrioid carcinoma, NOS	White	F	61	2A	0.51	517.2
93	Mixed cell adenocarcinoma	White	F	52	2A	0.56	11.2
94	Clear cell carcinoma	White	F	62	2B	0.52	110.8
95	Endometrioid adenocarcinoma, NOS	White	F	59	2B	0.72	133.0
96	Serous surface papillary carcinoma (C56.9)	White	F	65	2C	2.29	19.2
97	Papillary serous cystadenocarcinoma	White	F	76	3C	0.52	N/A

98	Serous adenocarcinoma, NOS (C56.9)	White	F	52	3C	0.56	836.6
99	Endometrioid adenocarcinoma, NOS	White	F	47	3C	1.22	160.4
100	Serous surface papillary carcinoma (C56.9)	White	F	54	3C	0.95	991.6
101	Mixed cell adenocarcinoma	White	F	50	3C	0.65	N/A
102	Carcinosarcoma, NOS	White	F	64	4	1.37	81.8
103	Serous surface papillary carcinoma	White	F	68	4	0.88	14.1
104	Serous surface papillary carcinoma	White	F	56	4	0.50	54.3
105	Mucinous adenocarcinoma	Black	F	60	4	0.59	N/A
106	Serous cystadenocarcinoma, NOS (C56.9)	White	F	65	4	1.28	6.4

*These normal controls are the same normal controls #1, #3-6, #8, #11-13, #16, #81, #83, #84, #88, #89, #92, #95-98, #100, #103, #106-109 in Table S1.

Supplementary Table S3. Characteristics of Prostate Cancer Patients and Controls

ID	Cancer Morphology	Race	Gender	Age	Stage	Exosomal TF-Ag-α level (A.U.)	Serum PSA level (ng/mL)
**Training set (30 normal controls and 40 cancer cases)**							
*1	Normal control	White	M	59		-0.27	N/A
*2	Normal control	White	M	76		0.13	N/A
*3	Normal control	White	M	52		-0.41	N/A
*4	Normal control	Black	M	60		-0.66	N/A
*5	Normal control	White	M	57		-0.03	N/A
*6	Normal control	White	M	69		0.38	N/A
*7	Normal control	Black	M	69		-0.96	N/A
*8	Normal control	White	M	62		-0.31	N/A
*9	Normal control	White	M	66		-0.12	N/A
*10	Normal control	White	M	51		-1.53	N/A
11	Benign prostatic hyperplasia (BPH)	White	M	65	Benign	-0.28	N/A
12	High-grade prostatic intraepithelial neoplasia (HGPIN) (PIN III)	White	M	64	Benign	-0.32	<0.03
13	Focal glandular hyperplasia	White	M	57	Benign	-0.26	N/A
14	HGPIN (PIN III)	White	M	63	Benign	-0.50	5.11
15	HGPIN (PIN III)	White	M	70	Benign	-0.90	31.69
16	Phyllodes tumor, benign	White	M	70	Benign	-0.39	<0.03
17	Prostatitis	White	M	55	Benign	0.40	N/A
18	HGPIN (PIN III)	White	M	60	Benign	-0.88	3.06
19	HGPIN (PIN III)	Black	M	72	Benign	-0.73	10.41
20	Focal glandular hyperplasia	White	M	64	Benign	-0.35	6.69
21	BPH	Unknown	M	75	Benign	-0.72	6.82
22	BPH	White	M	63	Benign	0.29	13.80
23	BPH	White	M	69	Benign	-1.36	0.96
24	BPH	White	M	60	Benign	-0.12	13.58
25	BPH	White	M	74	Benign	-0.57	N/A
26	BPH	White	M	53	Benign	-0.22	4.26
27	BPH	White	M	73	Benign	-0.77	N/A
28	HGPIN (PIN III)	White	M	59	Benign	0.36	0.80
29	Prostatitis	White	M	78	Benign	-0.76	3.64

30	BPH	White	M	60	Benign	-0.97	2.30
31	Adenocarcinoma, NOS	White	M	55	1	0.89	0.04
32	Adenocarcinoma, NOS	White	M	63	1	0.63	<0.04
33	Adenocarcinoma, NOS	Asian	M	71	1	0.89	<0.03
34	Adenocarcinoma, NOS	White	M	49	1	1.39	0.03
35	Acinar adenocarcinoma (C61.9 ONLY) (For prostate only, do not use 8550/3)	White	M	58	1	0.75	<0.04
36	Adenocarcinoma, NOS	White	M	75	1	1.26	<0.03
37	Adenocarcinoma, NOS	White	M	55	1	0.74	4.11
38	Adenocarcinoma, NOS	White	M	58	1	0.72	<0.03
39	Adenocarcinoma, NOS	White	M	65	1	1.30	<0.04
40	Adenocarcinoma, NOS	White	M	58	1	1.01	N/A
41	Adenocarcinoma, NOS	White	M	62	2	0.70	<0.03
42	Adenocarcinoma, NOS	White	M	51	2	0.80	N/A
43	Adenocarcinoma, NOS	White	M	54	2B	0.75	0.16
44	Adenocarcinoma, NOS	White	M	69	2B	0.87	<0.04
45	Adenocarcinoma, NOS	White	M	55	2B	1.00	<0.03
46	Adenocarcinoma, NOS	White	M	56	2B	1.69	<0.04
47	Acinar adenocarcinoma (C61.9 ONLY) (For prostate only, do not use 8550/3)	White	M	68	2B	0.81	<0.04
48	Adenocarcinoma, NOS	White	M	67	2B	1.19	N/A
49	Adenocarcinoma, NOS	White	M	59	2B	0.90	<0.03

50	Adenocarcinoma, NOS	White	M	51	2B	2.50	0.05
51	Adenocarcinoma, NOS	White	M	69	3	1.35	0.15
52	Adenocarcinoma, NOS	White	M	66	3	1.05	<0.03
53	Acinar adenocarcinoma (C61.9 ONLY) (For prostate only, do not use 8550/3)	White	M	50	3A	1.48	<0.04
54	Adenocarcinoma, NOS	White	M	60	3A	1.13	<0.04
55	Adenocarcinoma, NOS	White	M	62	3B	1.41	4.76
56	Adenocarcinoma, NOS	White	M	62	3B	1.25	14.82
57	Adenocarcinoma, NOS	White	M	52	3B	0.99	0.07
58	Adenocarcinoma, NOS	White	M	56	3B	1.32	39.57
59	Adenocarcinoma, NOS	White	M	62	3C	1.04	6.92
60	Adenocarcinoma, NOS	White	M	47	3C	1.06	1.78
61	Adenocarcinoma, NOS	White	M	45	4	1.25	323.39
62	Adenocarcinoma, NOS	Unknown	M	71	4B	1.07	189.44
63	Adenocarcinoma, NOS	White	M	81	4B	2.71	976.64
64	Adenocarcinoma, NOS	White	M	47	4B	1.09	3.34
65	Acinar adenocarcinoma	White	M	62	4A	0.57	0.17
66	Adenocarcinoma, NOS	White	M	58	4A	1.50	N/A
67	Adenocarcinoma, NOS	White	M	61	4A	1.03	7.97
68	Adenocarcinoma, NOS	White	M	67	4	0.62	4.49
69	Adenocarcinoma, NOS	White	M	58	4	0.67	N/A
70	Adenocarcinoma,	White	M	60	4	1.37	0.17

	NOS						
**Test set (13 normal controls and 20 cancer cases)**							
*71	Normal control (low risk)	White	M	54		0.07	N/A
*72	Normal control (low risk)	Black	M	58		-1.11	N/A
*73	Normal control (low risk)	Black	M	51		-0.81	N/A
*74	Normal control (low risk)	Asian	M	54		-1.23	N/A
*75	Normal control (low risk)	White	M	72		-0.63	N/A
*76	Normal control (low risk)	White	M	67		-1.39	N/A
*77	Normal control (low risk)	White	M	77		-0.71	N/A
*78	Normal control (low risk)	White	M	72		-1.43	N/A
*79	Normal control (low risk)	White	M	55		-0.74	N/A
*80	Normal control (low risk)	White	M	72		0.18	N/A
*81	Normal control (low risk)	White	M	72		-0.25	N/A
*82	Normal control (low risk)	White	M	61		-0.19	N/A
*83	Normal control (low risk)	White	M	55		0.07	N/A
84	Adenocarcinoma, NOS	White	M	57	1	0.65	<0.04
85	Adenocarcinoma, NOS	White	M	74	1	0.57	<0.03
86	Adenocarcinoma, NOS	White	M	71	1	0.82	<0.04
87	Acinar adenocarci-noma	Black	M	68	1	1.26	<0.04
88	Adenocarcinoma, NOS	White	M	61	1	1.11	0.17
89	Adenocarcinoma, NOS	White	M	59	2	2.03	N/A
90	Adenocarcinoma, NOS	White	M	56	2	1.74	0.03
91	Adenocarcinoma,	White	M	66	2B	0.72	N/A

	NOS						
92	Adenocarcinoma, NOS	White	M	57	2B	0.54	<0.04
93	Adenocarcinoma, NOS	Unknown	M	61	2B	1.49	N/A
94	Adenocarcinoma, NOS	Black	M	55	3	1.13	<0.04
95	Adenocarcinoma, NOS	White	M	59	3	0.86	N/A
96	Adenocarcinoma, NOS	Black	M	58	3B	0.49	<0.04
97	Adenocarcinoma, NOS	White	M	69	3B	0.86	<0.04
98	Adenocarcinoma, NOS	White	M	58	3C	0.52	<0.04
99	Infiltrating duct carcinoma, NOS	White	M	59	4	1.07	N/A
100	Adenocarcinoma, NOS	White	M	63	4	2.10	N/A
101	Adenocarcinoma, NOS	White	M	53	4	0.50	N/A
102	Adenocarcinoma, NOS	White	M	72	4	0.74	<0.04
103	Adenocarcinoma, NOS	White	M	58	4	0.79	<0.03

*These normal controls are the same normal controls #2, #7, #9, #10, #14, #15, #17-20, #82, #85-87, #90, #91, #93, #94, #99, #101, #102, #104, #105in Table S1.

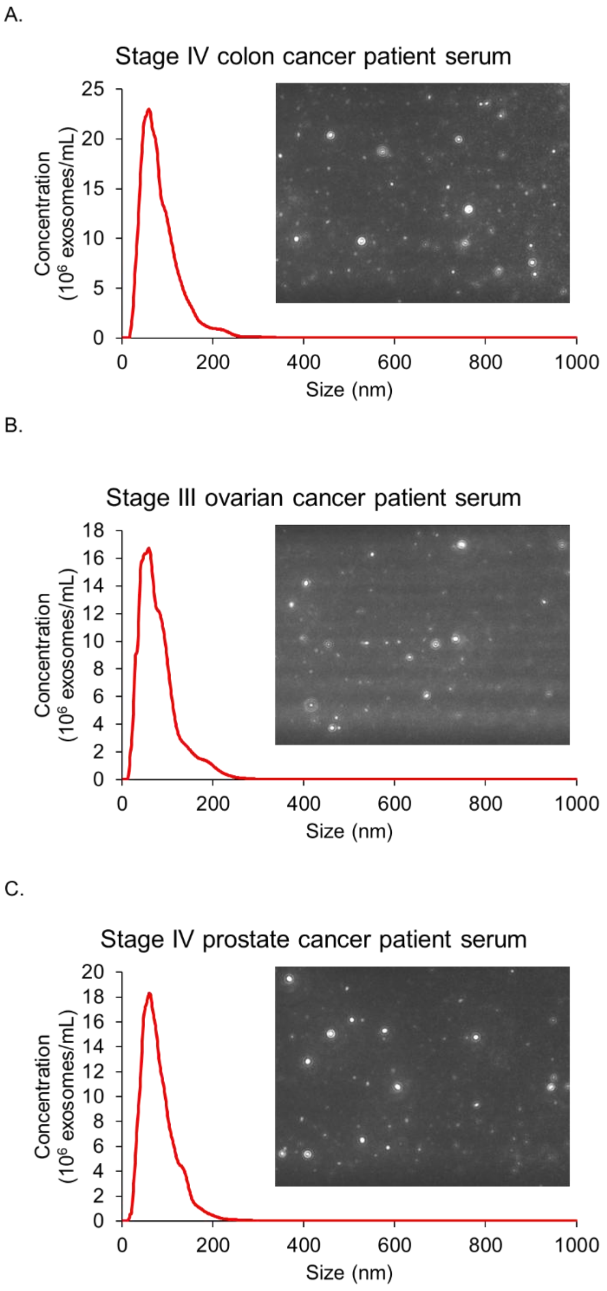

Supplementary Figure S1. Characterization of exosomes isolated from patient serum samples via nanoparticle tracking analysis (NTA). (A) Representative size distribution curve of exosomes isolated from 100 uL serum of a stage IV colon cancer patient (10,000X dilution). The mean diameter of exosomes was 85.56 nm (n = 3). (B) Representative size distribution of exosomes isolated from 100 uL serum of a stage III ovarian cancer patient (1,000X dilution). The mean diameter of exosomes was 80.52 nm (n = 3). (C) Representative size distribution of exosomes isolated from 100 uL serum of a stage IV prostate cancer patient (10,000X dilution). The mean diameter of exosomes was 81.16 nm (n = 3). Insert: Screen-shot from the recorded video of exosomes during NTA.

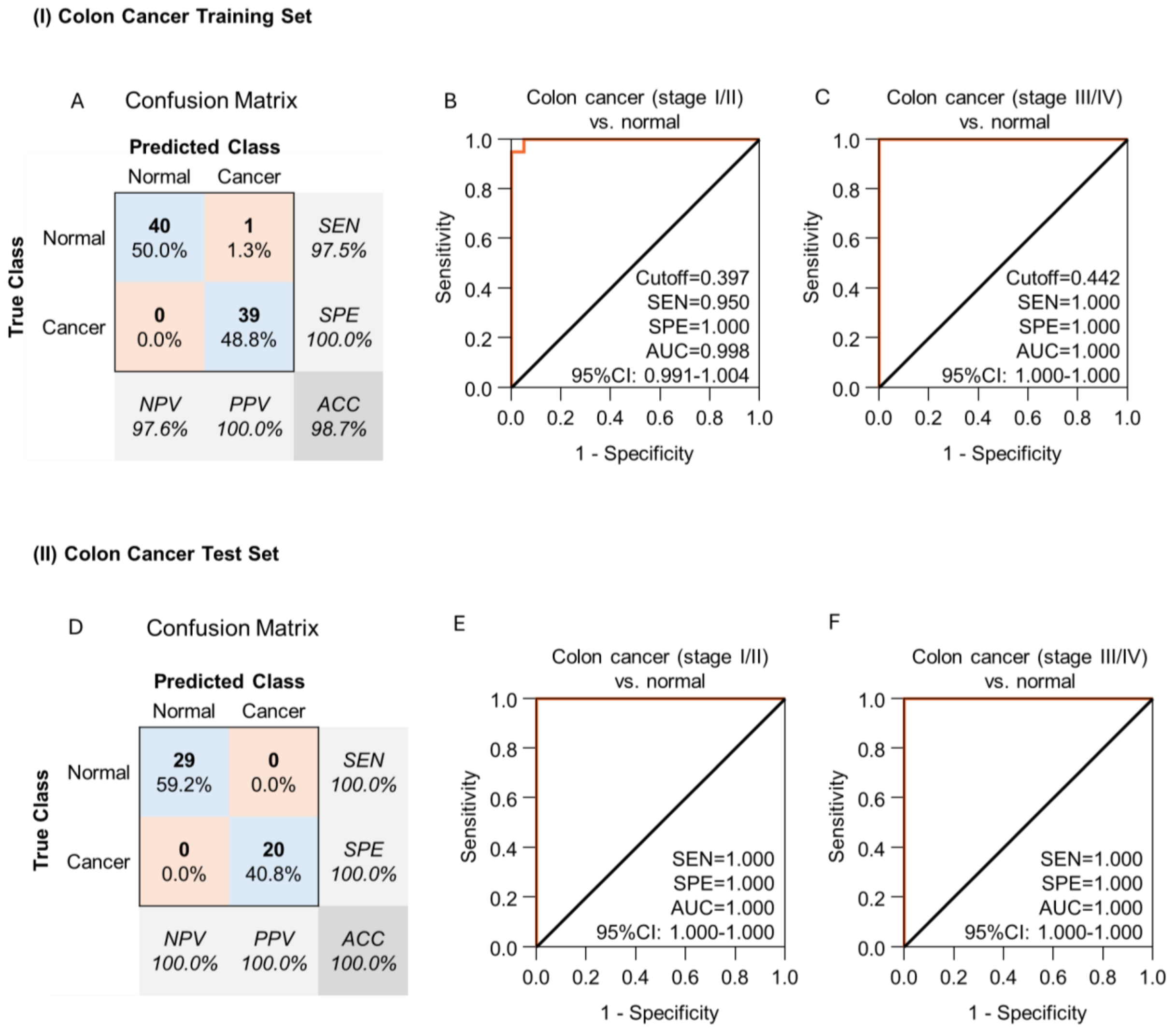

Supplementary Figure S2. Evaluation of exosomal TF-Ag-α for colon cancer diagnosis using a training set and an independent test set. (A) Confusion matrix showed the diagnostic performance of exosomal TF-Ag-α in colon cancer based on the training set which included 40 colon cancer patients (stage I/II: n = 20; stage III/IV: n = 20) and 40 normal controls (low risk: n = 20; benign conditions at high risk of cancer: n = 20). (B) ROC curve analysis comparing early-stage colon cancer patients (Stage I/II, n = 20) vs. normal controls (n = 40) in the training set. (C) ROC curve analysis comparing late-stage colon cancer patients (Stage III/IV, n = 20) vs. normal controls (n = 40) in the training set. (D) Confusion matrix showed the diagnostic performance of exosomal TF-Ag-α based on the test set which included 20 colon cancer patients (stage I/II: n = 10; stage III/IV: n = 10) and 29 normal controls. (E) ROC curve analysis comparing early-stage colon cancer patients (Stage I/II, n = 10) vs. normal controls (n = 29) in the test set. (F) ROC curve analysis comparing late-stage colon cancer patients (Stage III/IV, n = 10) vs. normal controls (n = 29) in the test set. (SEN: sensitivity, SPE: specificity, NPV: negative predictive value; PPV: positive predictive value, ACC: overall accuracy, AUC: area under the curve.)

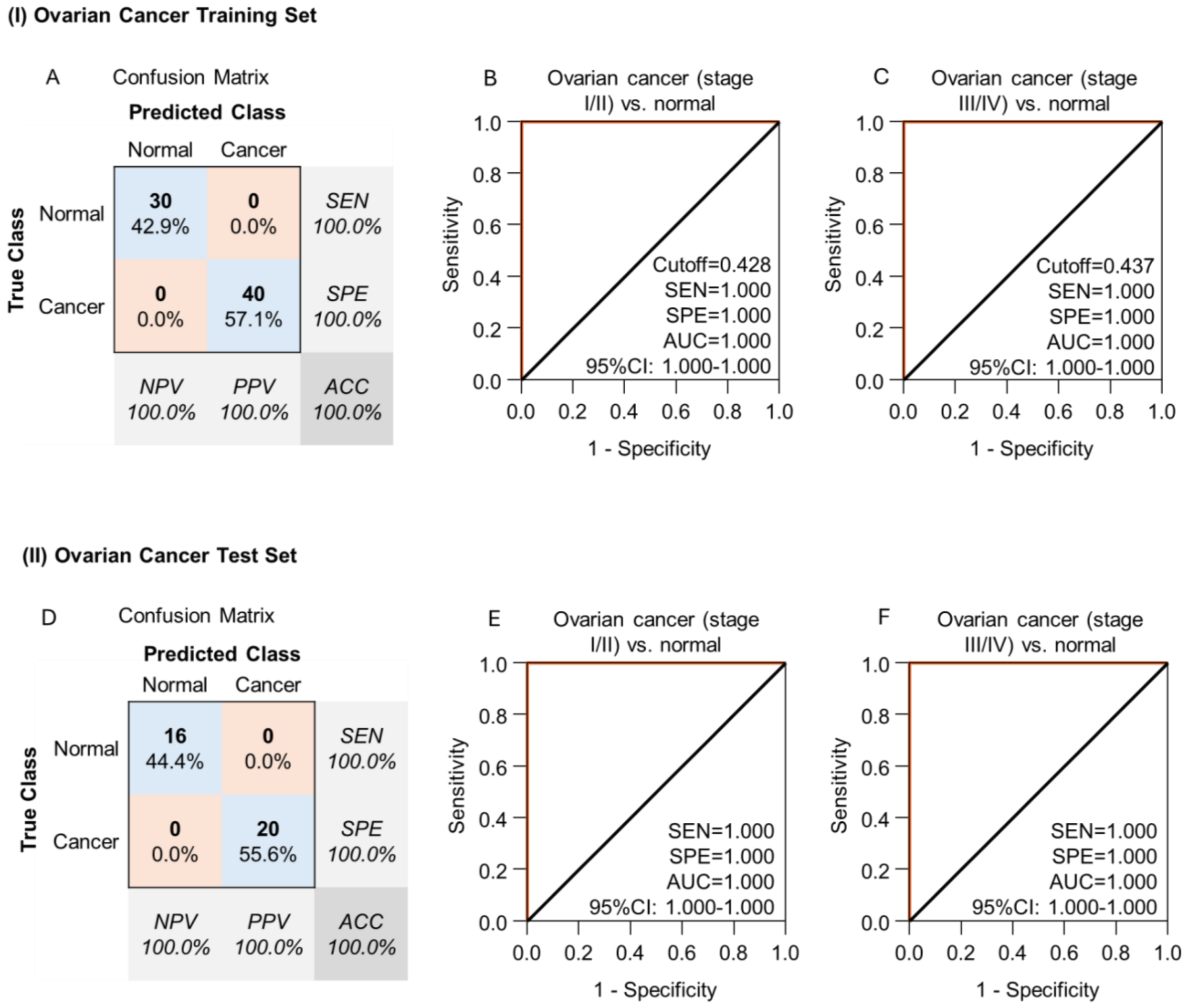

Supplementary Figure S3. Evaluation of exosomal TF-Ag-α for ovarian cancer diagnosis using a training set and an independent test set. (A) Confusion matrix showed the diagnostic performance of exosomal TF-Ag-α in ovarian cancer based on the training set which included 40 ovarian cancer patients (stage I/II: n = 20; stage III/IV: n = 20) and 30 normal controls (low risk: n = 10; benign conditions at high risk of cancer: n = 20). (B) ROC curve analysis comparing early-stage ovarian cancer patients (Stage I/II, n = 20) vs. normal controls (n = 30) in the training set. (C) ROC curve analysis comparing late-stage ovarian cancer patients (Stage III/IV, n = 20) vs. normal controls (n = 30) in the training set. (D) Confusion matrix showed the diagnostic performance of exosomal TF-Ag-α based on the test set which included 20 ovarian cancer patients (stage I/II: n = 10; stage III/IV: n = 10) and 16 normal controls. (E) ROC curve analysis comparing early-stage ovarian cancer patients (Stage I/II, n = 10) vs. normal controls (n = 16) in the test set. (F) ROC curve analysis comparing late-stage ovarian cancer patients (Stage III/IV, n = 10) vs. normal controls (n = 16) in the test set. (SEN: sensitivity, SPE: specificity, NPV: negative predictive value; PPV: positive predictive value, ACC: overall accuracy, AUC: area under the curve.)

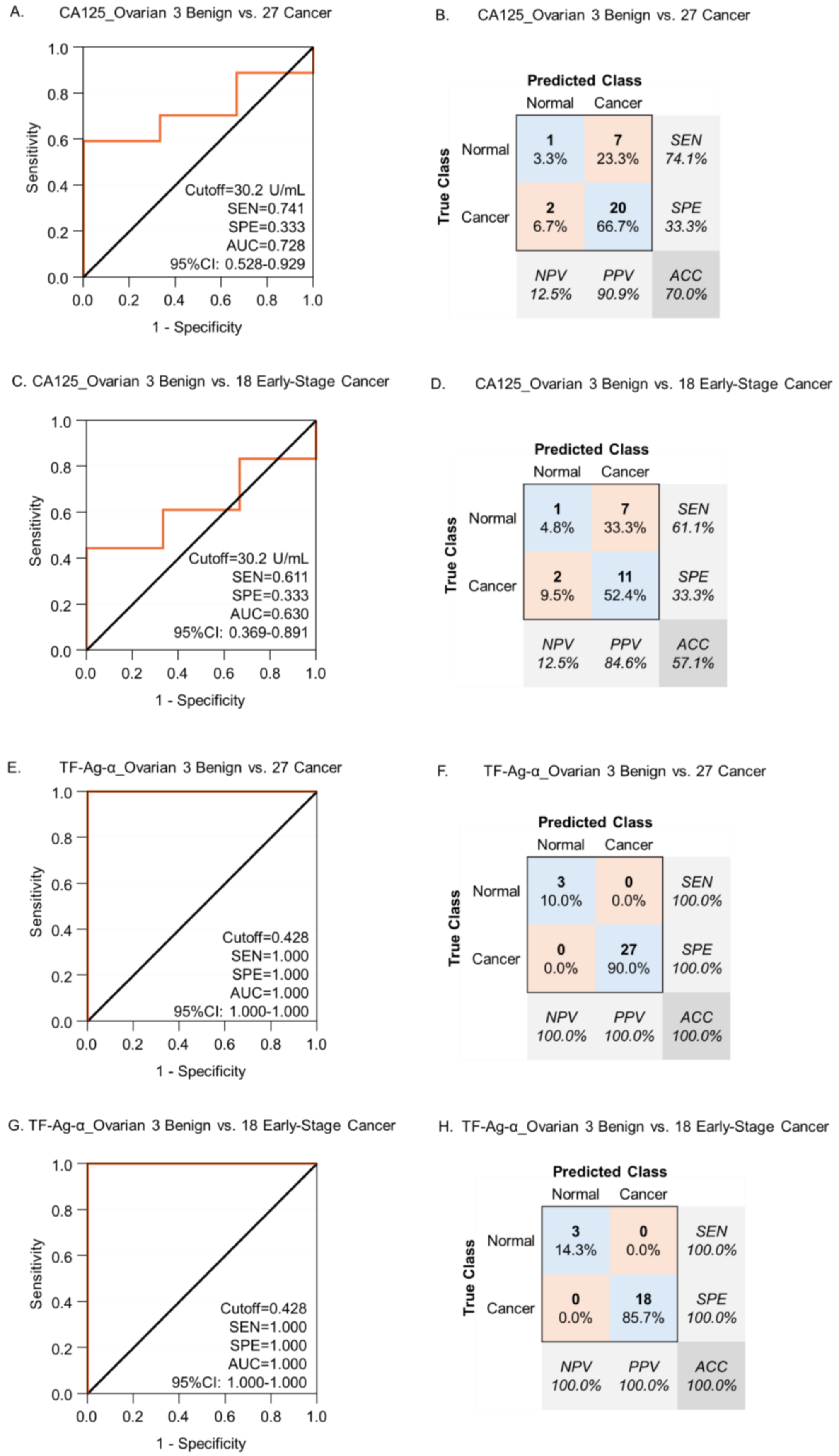

Supplementary Figure S4. Diagnostic performance comparison between CA125 and exosomal TF-Ag-α in ovarian cancer. Pre-treatment CA125 data were available for a subset of ovarian cancer patients (n = 27) and patients with benign

conditions (n = 3), with blood samples collected within 100 days of those used for exosomal TF-Ag-α analysis to maintain temporal and analytical consistency. (A) ROC curve analysis comparing patients with ovarian cancer (n = 27, stage I-IV) vs. benign conditions (n = 3) using CA125 as the biomarker. (B) The confusion matrix illustrated the diagnostic performance of CA125 based on 27 ovarian cancer patients and 3 patients with benign conditions. (C) ROC curve analysis comparing patients with early-stage ovarian cancer (n = 18, stage I-II) vs. benign conditions (n = 3) using CA125 as the biomarker. (D) Confusion matrix showed diagnostic performance of CA125 based on 18 early-stage ovarian cancer patients and 3 patients with benign conditions. (E) ROC curve analysis comparing patients with ovarian cancer (n = 27, stage I-IV) vs. benign conditions (n = 3) using exosomal TF-Ag-α as the biomarker. (F) Confusion matrix showed diagnostic performance of exosomal TF-Ag-α based on 27 ovarian cancer patients and 3 patients with benign conditions. (G) ROC curve analysis comparing patients with early-stage ovarian cancer (n = 18, stage I-II) vs. benign conditions (n = 3) using exosomal TF-Ag-α as the biomarker. (H) Confusion matrix showed diagnostic performance of exosomal TF-Ag-α based on 18 early-stage ovarian cancer patients and 3 patients with benign conditions.

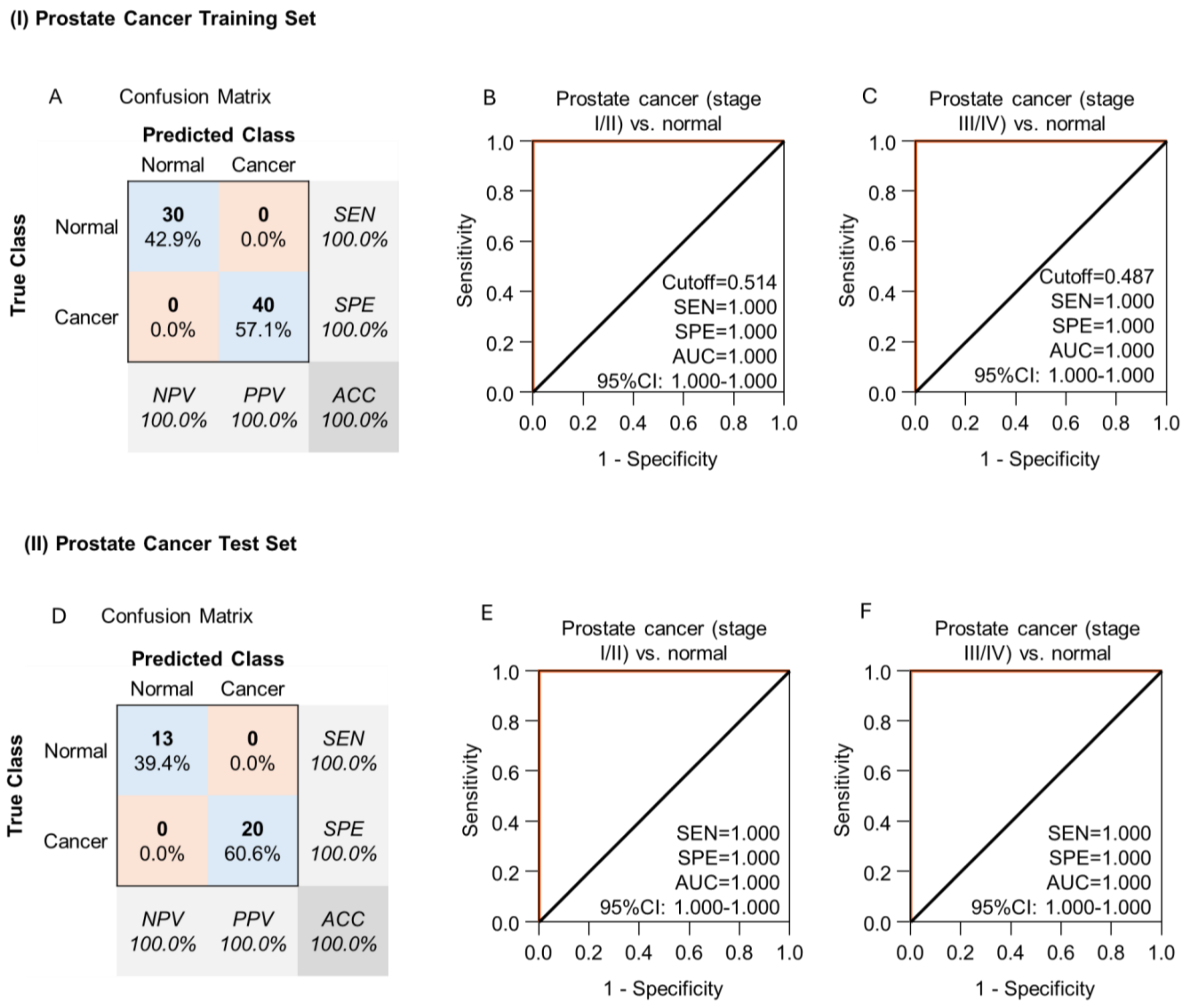

Supplementary Figure S5. Evaluation of exosomal TF-Ag-α for prostate cancer diagnosis using a training set and an independent test set. (A) Confusion matrix showed the diagnostic performance of exosomal TF-Ag-α in prostate cancer based on the training set which included 40 prostate cancer patients (stage I/II: n = 20; stage III/IV: n = 20) and 30 normal controls (low risk: n = 10; benign conditions at high risk of cancer: n = 20). (B) ROC curve analysis comparing early-stage prostate cancer patients (Stage I/II, n = 20) vs. normal controls (n = 30) in the training set. (C) ROC curve analysis comparing late-stage prostate cancer patients (Stage III/IV, n = 20) vs. normal controls (n = 30) in the training set. (D) Confusion matrix showed the diagnostic performance of exosomal TF-Ag-α based on the test set which included 20 prostate cancer patients (stage I/II: n = 10; stage III/IV: n = 10) and 13 normal controls. (E) ROC curve analysis comparing early-stage prostate cancer patients (Stage I/II, n = 10) vs. normal controls (n = 13) in the test set. (F) ROC curve analysis comparing late-stage prostate cancer patients (Stage III/IV, n = 10) vs. normal controls (n = 13) in the test set. (SEN: sensitivity, SPE: specificity, NPV: negative predictive value; PPV: positive predictive value, ACC: overall accuracy, AUC: area under the curve.)

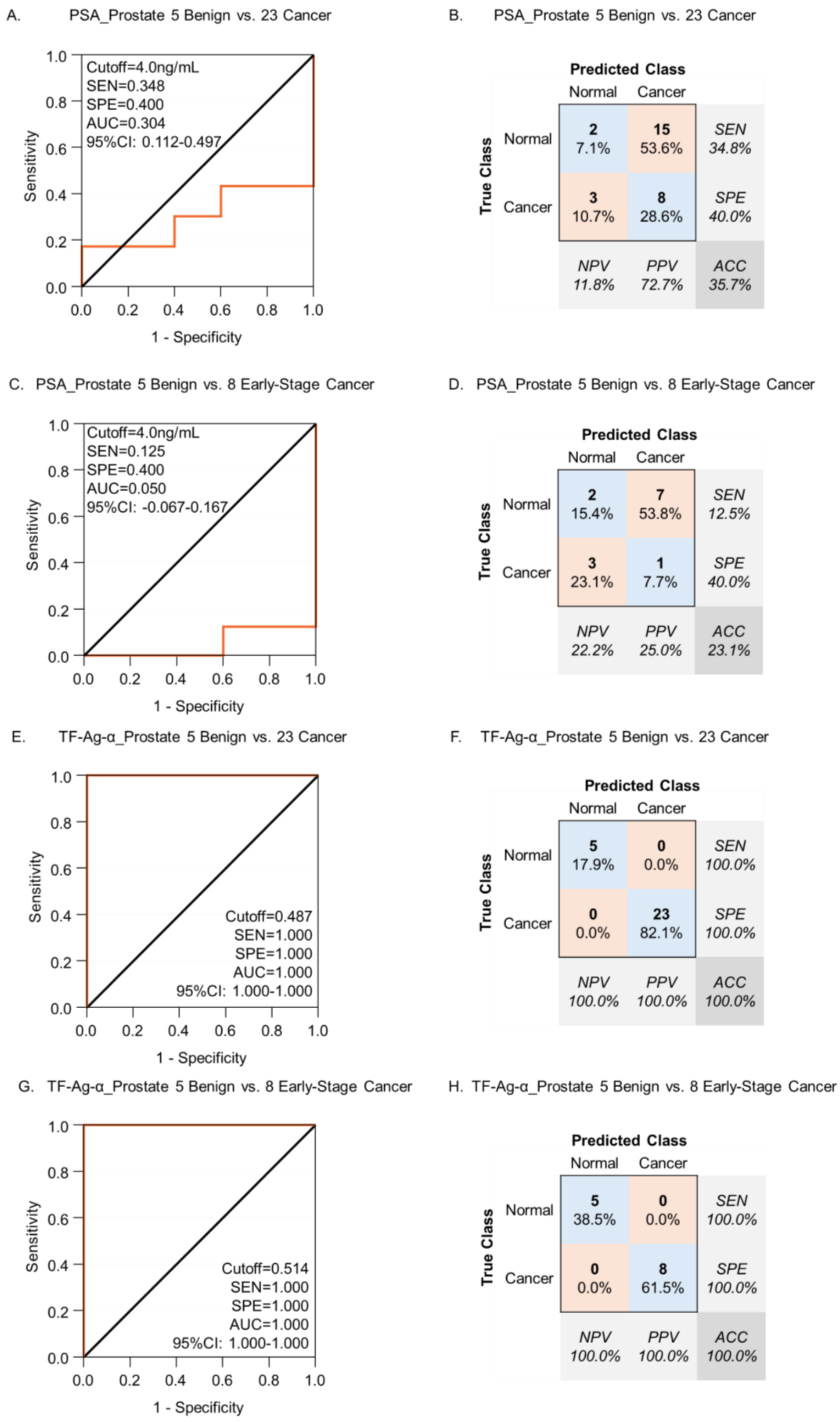

Supplementary Figure S6. Diagnostic performance comparison between PSA and exosomal TF-Ag-α in prostate cancer. Pre-treatment PSA data were available for a subset of prostate cancer patients (n = 23) and patients with

benign conditions (n = 5), with blood samples collected within 100 days of those used for exosomal TF-Ag-α analysis to maintain temporal and analytical consistency. (A) ROC curve analysis comparing patients with prostate cancer (n = 23, stage I-IV) vs. benign conditions (n = 5) using PSA as the biomarker. (B) The confusion matrix illustrated the diagnostic performance of PSA based on 23 prostate cancer patients and 5 patients with benign conditions. (C) ROC curve analysis comparing patients with early-stage prostate cancer (n = 8, stage I-II) vs. benign conditions (n = 5) using PSA as the biomarker. (D) Confusion matrix showed diagnostic performance of PSA based on 8 early-stage prostate cancer patients and 8 patients with benign conditions. (E) ROC curve analysis comparing patients with prostate cancer (n = 23, stage I-IV) vs. benign conditions (n = 5) using exosomal TF-Ag-α as the biomarker. (F) Confusion matrix showed diagnostic performance of exosomal TF-Ag-α based on 23 prostate cancer patients and 5 patients with benign conditions. (G) ROC curve analysis comparing patients with early-stage prostate cancer (n = 8, stage I-II) vs. benign conditions (n = 5) using exosomal TF-Ag-α as the biomarker. (H) Confusion matrix showed diagnostic performance of exosomal TF-Ag-α based on 8 early-stage prostate cancer patients and 5 patients with benign conditions.

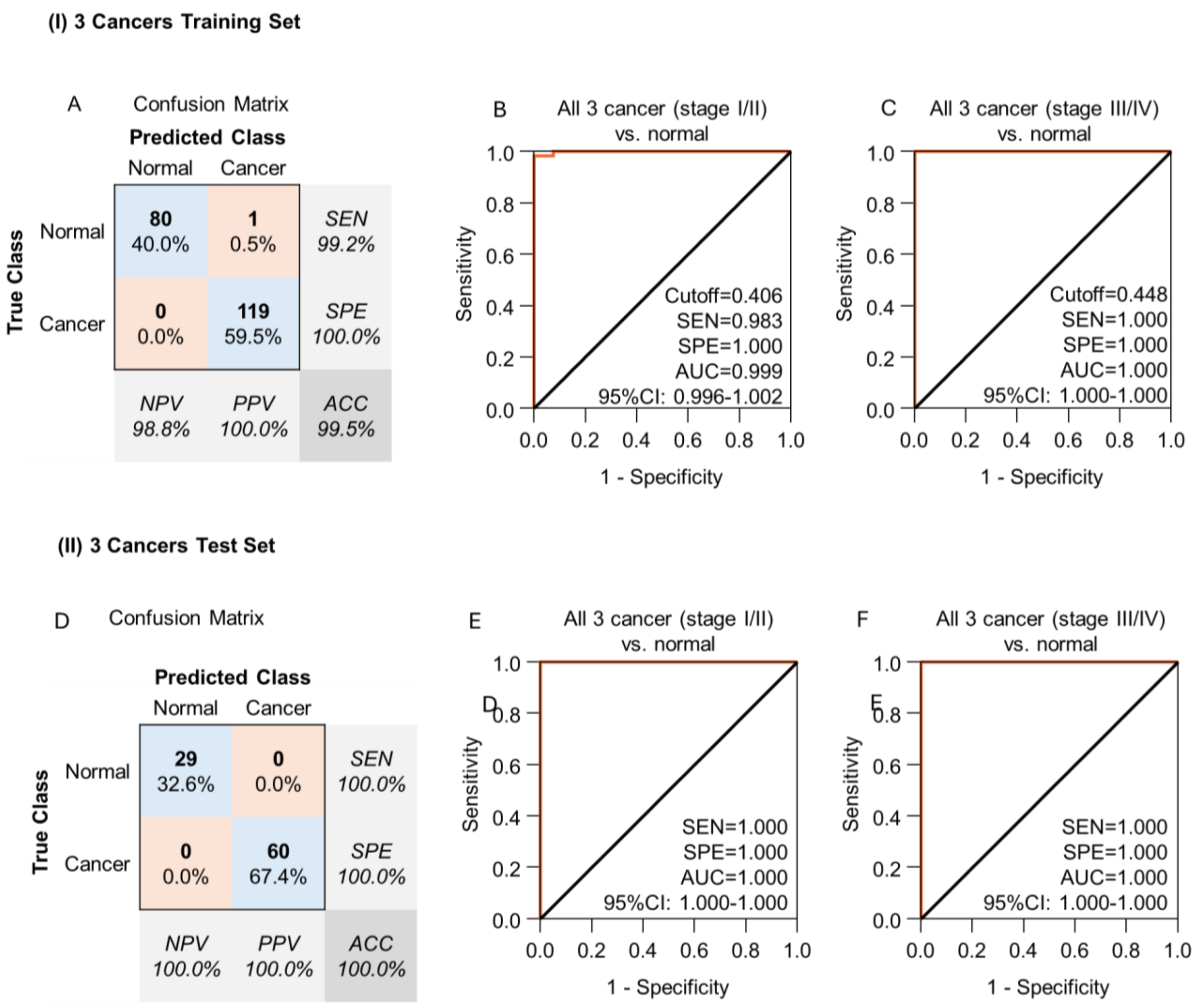

Supplementary Figure S7. Evaluation of exosomal TF-Ag-α in three cancer diagnosis using the pooled training sets and independent test sets (colon, ovarian and prostate cancers). (A) Confusion matrix showed the diagnostic performance of exosomal TF-Ag-α in 3 cancers based on the pooled training sets which included 120 cancer patients (stage I/II: n = 60; stage III/IV: n = 60) and 80 normal controls (low risk: n = 20; benign conditions at high risk of cancer: n = 60). (B) ROC curve analysis comparing early-stage cancer patients (Stage I/II, n = 60) vs. normal controls (n = 80) in the training set. (C) ROC curve analysis comparing late-stage cancer patients (Stage III/IV, n = 60) vs. normal controls (n = 80) in the training set. (D) Confusion matrix showed the diagnostic performance of exosomal TF-Ag-α based on the pooled test sets which included 60 cancer patients (stage I/II: n = 30; stage III/IV: n = 30) and 29 normal controls. (E) ROC curve analysis comparing early-stage cancer patients (Stage I/II, n = 30) vs. normal controls (n = 29) in the test set. (F) ROC curve analysis comparing late-stage cancer patients (Stage III/IV, n = 30) vs. normal controls (n = 29) in the test set. (SEN: sensitivity, SPE: specificity, NPV: negative predictive value; PPV: positive predictive value, ACC: overall accuracy, AUC: area under the curve.)

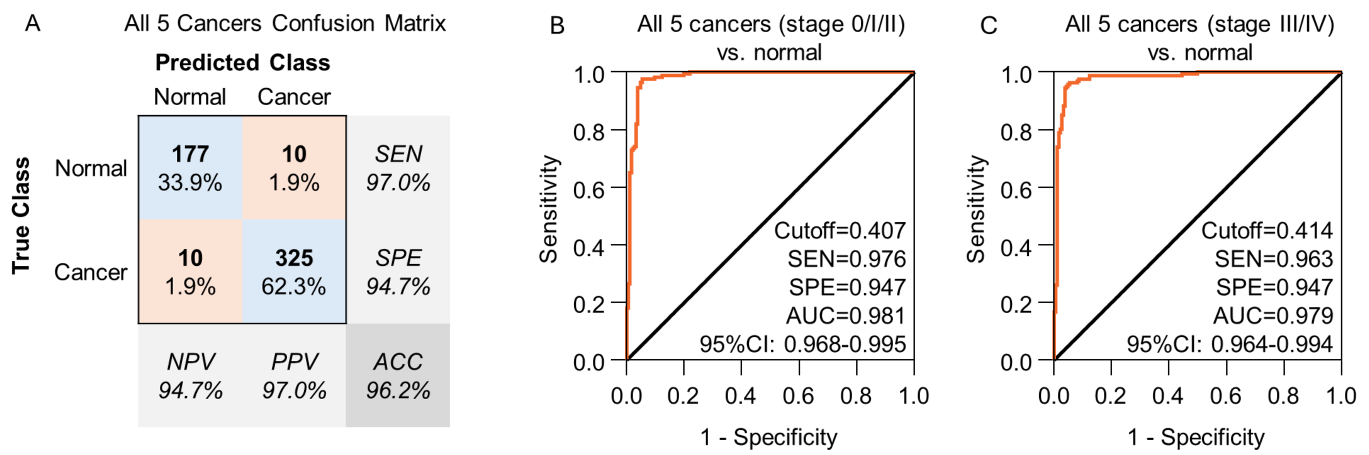

Supplementary Figure S8. Evaluation of exosomal TF-Ag-α in five cancer diagnosis (colon, ovarian, prostate, lung and breast cancers). (A) Confusion matrix showed the diagnostic performance of exosomal TF-Ag-α in 5 cancers based on all patient data which included 335 cancer patients (stage 0/I/II: n = 174; stage III/IV: n = 161) and 187 normal controls (low risk: n = 89; benign conditions at high risk of cancer: n = 98). (B) ROC curve analysis comparing early-stage cancer patients (Stage I/II, n = 174) vs. normal controls (n = 187). (C) ROC curve analysis comparing late-stage cancer patients (Stage III/IV, n = 161) vs. normal controls (n = 187). (SEN: sensitivity, SPE: specificity, NPV: negative predictive value; PPV: positive predictive value, ACC: overall accuracy, AUC: area under the curve.)

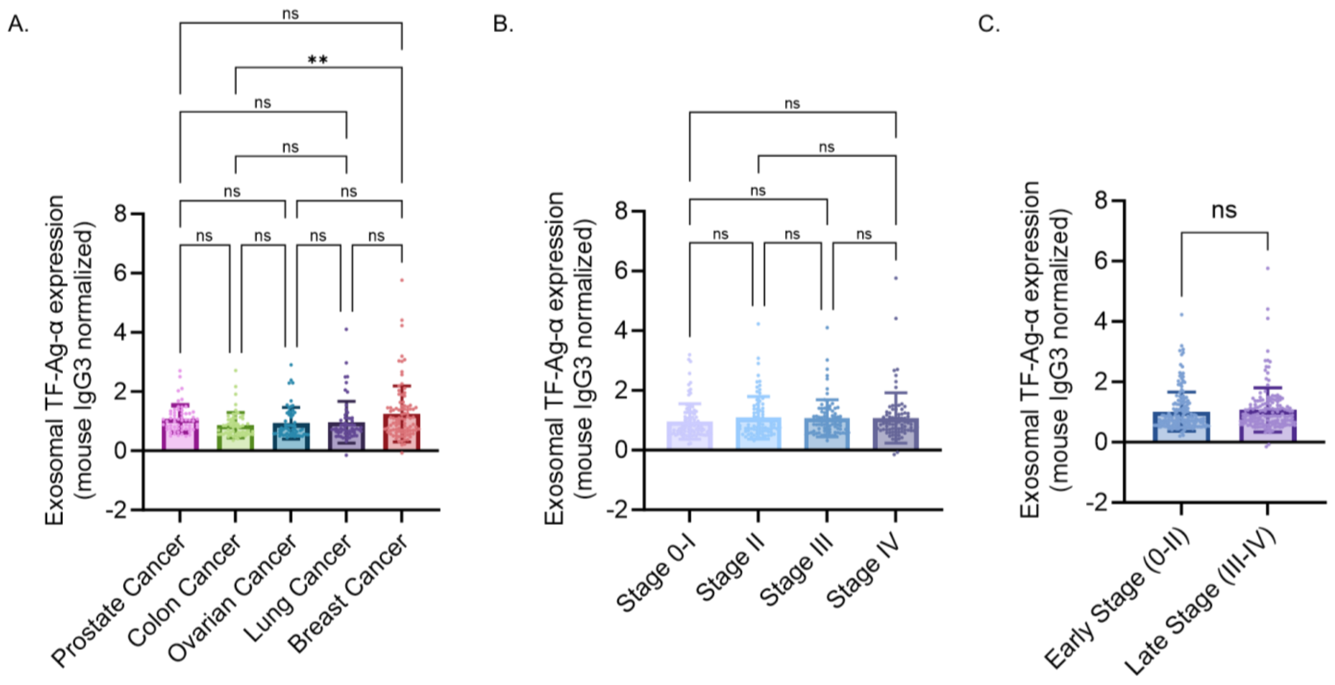

Supplementary Figure S9. Exosomal TF-Ag-α levels remain largely consistent across cancer types and stages. One-way ANOVA analysis of exosomal TF-Ag-α levels across cancer types and stages in five cancer types (colon, ovarian, prostate, lung and breast). (A) A Brown-Forsythe and Welch-corrected one-way ANOVA revealed significant differences in exosomal TF-Ag-α levels among the five cancer types (F (4.000, 160.9) = 3.728, p = 0.0063, **; F*(4.000, 286.8) = 4.381, p = 0.0019, **). Post-hoc Games-Howell comparisons indicated that breast cancer patients had significantly higher exosomal TF-Ag-α levels than colon cancer patients (p = 0.0071, **), while all other pairwise differences were not significant (ns). (B) A Brown-Forsythe and Welch-corrected one-way ANOVA showed no significant differences in exosomal TF-Ag-α levels among cancer stages (F (3.000, 177.6) = 0.8260, p = 0.4811; F*(3.000, 289.7) = 0.6793, p = 0.5653). Post-hoc Games-Howell comparisons showed no significant pairwise differences. (C) A Welch's unpaired t-test comparing early-stage (0–II) and late-stage (III–IV) cancers showed no significant difference in exosomal TF-Ag-α levels (P = 0.4344, ns).
